# Machine Learning on Systematically Curated Data Reveals Key Determinants of Magnetic Hyperthermia Performance

**DOI:** 10.1002/smll.202510453

**Published:** 2026-01-30

**Authors:** Edgar Régulo Vega‐Carrasco, Shaquib Rahman Ansari, Jiaxi Zhao, Yael del Carmen Suárez‐López, Per Larsson, Alexandra Teleki

**Affiliations:** ^1^ Department of Pharmacy Science for Life Laboratory Uppsala University Uppsala Sweden; ^2^ Department of Pharmacy Uppsala University Uppsala Sweden

**Keywords:** Bayesian optimization, CatBoost, conformal prediction, feature importance, machine learning, magnetic hyperthermia, SHAP analysis, specific absorption rate, superparamagnetic iron oxide nanoparticles

## Abstract

Accurate prediction of the specific absorption rate (SAR) of superparamagnetic iron oxide nanoparticles (SPIONs) is critical for optimizing their performance in magnetic hyperthermia applications. This study presents the development of a predictive model for SAR using advanced machine learning techniques and a systematically curated dataset comprising 1850 entries from 84 published studies, capturing 30 predictive features related to SPION properties and experimental parameters. Twelve machine learning algorithms were evaluated and optimized using Bayesian hyperparameter tuning. The CatBoost algorithm emerged as the top‐performing model (*R* ^2^ = 0.98) with the lowest prediction errors. Shapley additive explanation analysis revealed alternating magnetic field amplitude and frequency as the most influential factors determining SAR, followed by SPION concentration and core surface area. Model reliability was confirmed through conformal prediction, providing a prediction interval of ±62 W g^−1^. Validation using an independent dataset of SPIONs with varying sizes (7–30 nm) and dopants (Zn, Mn, Mg, Co) demonstrated strong predictive performance for small nanoparticles (≈7 nm), with increased variability for larger particles. These findings demonstrate that advanced machine learning models enable accurate SAR prediction and provide critical insights into nanoparticle design, supporting the systematic optimization of SPIONs for clinical magnetic hyperthermia applications.

## Graphical abstract

This study presents a machine‐learning (ML) framework to predict the specific absorption rate (SAR) of superparamagnetic iron oxide nanoparticles (SPIONs) for magnetic hyperthermia. A curated dataset comprising 30 intrinsic and extrinsic features revealed strong nonlinear dependencies. Twelve ML models were optimized using Bayesian methods, with CatBoost emerging as the best‐performing algorithm. Field amplitude and frequency, concentration, and core surface area were key predictors.

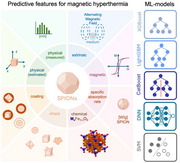

## 1 Introduction

Magnetic hyperthermia is a promising therapeutic technique used in clinical practice as a complementary treatment with chemotherapy against glioblastoma [1, 2]. Further, preclinical data suggest that it holds significant potential for a broader range of biomedical applications, such as synergistic elimination of bacterial biofilms [3] and controlled drug delivery for enhanced multimodal cancer treatment [4]. Magnetic hyperthermia involves the administration of superparamagnetic iron oxide nanoparticles (SPIONs) to the cancerous tissue, followed by the application of an alternating magnetic field (AMF) [5, 6, 7]. Under AMF, SPIONs dissipate heat, which causes tumor cells to shrink or lose vital functions owing to their higher thermal sensitivity compared to healthy tissue [6, 8]. The efficient delivery of SPIONs to deep‐seated and poorly accessible tumors can also facilitate minimally invasive therapy [9].

SPIONs are composed of magnetite (Fe_3_O_4_) or maghemite (γ‐Fe_2_O_3_) and typically have an average core diameter less than 20–25 nm [5, 10]. They do not retain magnetization in the absence of a magnetic field [6, 10], are efficiently cleared through the endogenous iron metabolism pathway, and exhibit low toxicity, thus showing excellent biocompatibility [9]. The clinical potential of SPIONs for magnetic hyperthermia is reflected in their approval by the European Medicines Agency and the United States Food and Drug Administration for clinical‐stage investigations in treating pancreatic and brain tumors [5]. Nonetheless, the therapeutic efficacy of magnetic hyperthermia and its broader applicability mainly rely on the heating efficiency of SPIONs [11]. Achieving high heating efficiency would enable low nanoparticle doses and milder AMF parameters, enhancing both safety and efficacy.

The heating efficiency of SPIONs can be assessed by the specific absorption rate (SAR), which measures the hyperthermia performance in terms of heating power generated per unit mass of nanoparticles [12]. SAR is influenced by both, intrinsic parameters such as nanoparticle size, shape, size distribution, chemical composition, magnetic properties, surface coating chemistry [13, 14, 15, 16, 17, 18, 19], and extrinsic parameters including AMF amplitude, AMF frequency, and the viscosity of the surrounding medium [20, 21, 22].

Controlling the size and morphology of SPIONs is critical to improve their hyperthermia performance [23, 24, 25, 26, 27]. A growing number of studies have reported the use of metal dopants such as Mn, Zn, Co, Ni, or Mg, to enhance SPIONs' hyperthermia performance in biomedical applications [28]. The incorporation of dopants into the iron oxide crystal lattice can alter the SPIONs' magnetic properties and increase their SAR [29, 30, 31, 32, 33].

Although doping offers a versatile route to tune hyperthermia performance, maintaining precise stoichiometry is challenging, as minor deviations in dopant levels can reduce performance [34, 35, 36, 37]. These complex dependencies complicate the efforts to predict or maximize SAR through empirical methods alone. Additionally, the parallel exploration of diverse nanoparticle properties also remains challenging due to the difficulty of synthesizing nanoparticle libraries with well‐defined, reproducible, and precisely controlled features [38].

Furthermore, the nanoparticle synthesis process itself adds further complexity, introducing batch‐to‐batch variability, poor crystallinity, and crystal structural defects [39]. Although mathematical models such as the linear response theory [40] and the Stoner‐Wohlfarth model [41] can be used to estimate SAR, these approaches do not account for intrinsic nanoparticle features such as size distribution, surface coating, chemical composition, and shape anisotropy. Therefore, there is a need for systematic, data‐driven approaches that can account for the numerous and complex feature interactions to accurately predict the heating performance of SPIONs.

Machine learning (ML)‐assisted approaches are known for their ability to handle large datasets and accelerate the development and optimization of nanomaterials [42]. Machine learning methods have been widely applied across the fields of chemistry and materials engineering to predict nanoparticle properties [43, 44, 45, 46]. Previously, ML models have successfully developed design rules for synthesizing titanium dioxide and iron oxide nanoparticles [43, 46]. This approach enabled the prediction of key synthesis outcomes, including nanoparticle size, shape, polydispersity, and phase. Furthermore, a similar approach was applied using a simulated dataset to predict the magnetic properties of iron oxide nanoparticles for hyperthermia applications [44]. However, reliance on simulated datasets limits model generalizability, while the diverse experimental conditions reported across the literature further complicate direct model comparison and transferability across studies.

A recent study reported the use of tree‐based ML models on literature‐derived experimental data to predict the SAR relying on physical, magnetic, and AMF features [45]. However, that approach did not consider key predictive features such as the size distribution of SPIONs or the direct influence of dopants, such as Mg, Zn, Co, and Mn. These physicochemical properties are important design parameters that should be considered when optimizing SPIONS with therapeutic applications [39, 47]. Therefore, establishing a systematic approach to SPION development is crucial for the successful translation of this promising therapeutic technique into clinical practice. To address this need, we have developed a ML approach that supports the design and optimization of SPIONs.

This study aims to develop ML models for predicting the magnetic hyperthermia performance of SPIONs based on their physical, chemical, and magnetic properties. To accomplish this objective, a novel workflow was developed by integrating semi‐automated dataset building, ML model building based on Bayesian hyperparameter optimization, comparative absolute error box‐plot analysis, agglomerative hierarchical clustering analysis, permutation importance, and conformal prediction assessment. Specifically, this data‐driven workflow integrates systematic review guidelines [48] and natural language processing (NLP) techniques to build a literature‐derived dataset that describes SPION hyperthermia performance.

This unique dataset compiles information from 1850 SAR measurements, along with intrinsic nanoparticle properties and extrinsic experimental parameters from published scientific literature. The unique dataset and the integrated workflow were employed to evaluate 12 ML algorithms, build multiple models, fine‐tune hyperparameters, and ultimately select the model with the highest predictive performance. Finally, a feature importance analysis was conducted on the top‐performing model to explain the impact of physical, chemical, and magnetic properties on the SAR performance. In summary, our study reports a unique ML approach to support the design of SPIONs aiming to enhance their hyperthermia performance.

## 2 Results and Discussion

### 2.1 Semi‐Automated Dataset Building

**FIGURE 1:**
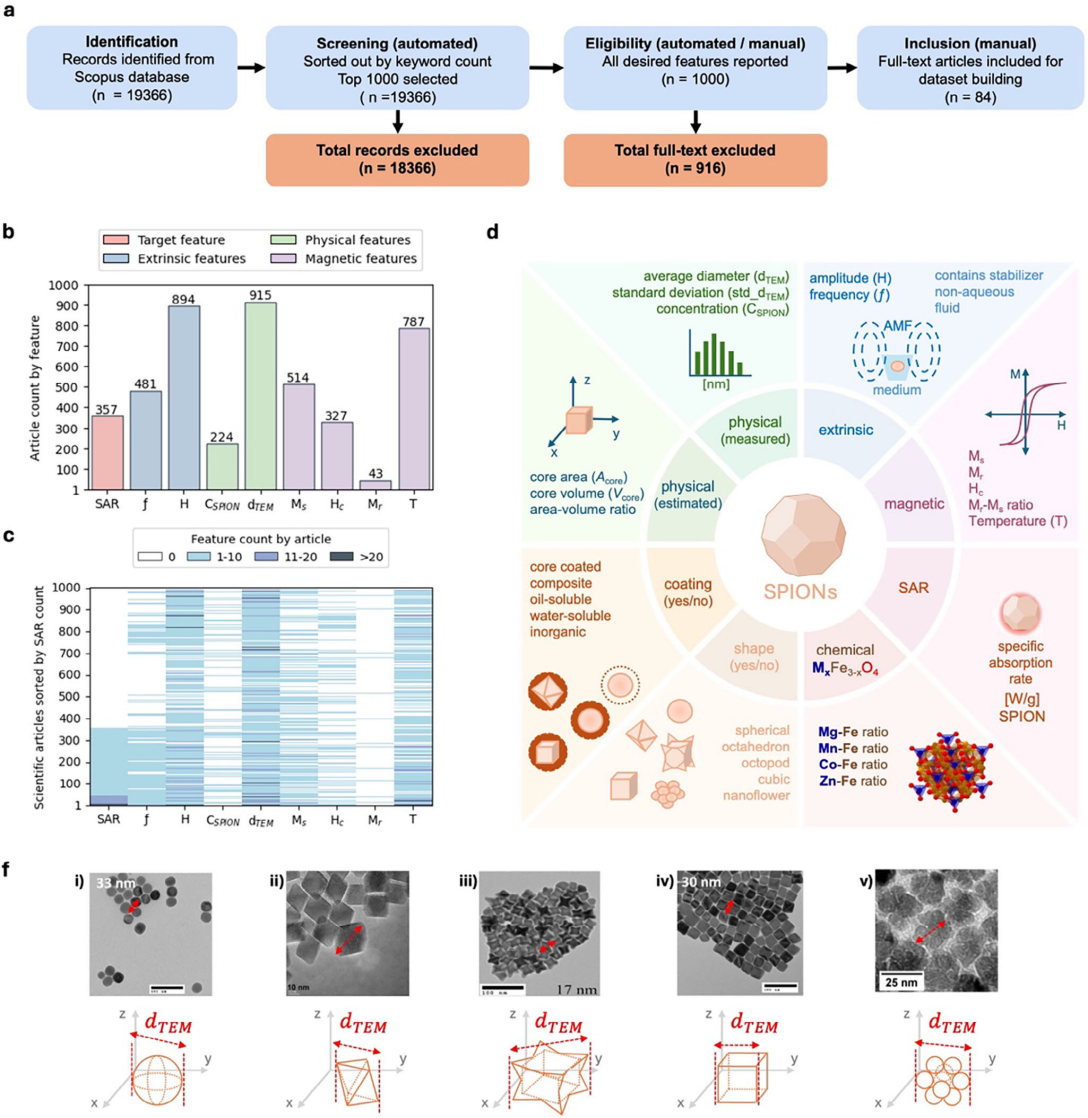
Dataset building workflow from semiautomated datapoint extraction to predictive feature identification, distribution analysis, and graphical stratification. (a) Flow diagram detailing the methodology applied to include scientific articles in the database. (b) Bar plot showing the number of scientific articles reporting specific magnetic or physical features. (c) Heatmap showing scientific articles sorted by the count of the specific absorption rate (SAR) along with magnetic and physical features. (d) Schematic representation of the predictive features of SPIONs in relation to SAR, integrating their physical, magnetic, chemical, and morphological properties. (f) Scheme detailing assumptions made to calculate the nanoparticle core volume and core surface area. Sphere and cubic nanoparticles are detailed in subpanels (i) and (iv), respectively. Nominal *d*_TEM_ values reported as 33 and 30 nm in micrograph (i) and (iv), respectively; adapted with permission from Nemati et al. [23], Copyright (2018), American Chemical Society. Octahedron nanoparticles are depicted in subpanel (ii); adapted with permission from Jovic et al. [32], Copyright (2020), IOP Publishing. Octopod nanoparticles are described in subpanel (iii). Nominal *d*_TEM_ values reported as 17 nm in micrograph (iii); adapted with permission from Nemati et al. [68], Copyright (2016), American Chemical Society. Nanoflowers are detailed in subpanel (iv); adapted with permission from Hemery et al. [59], Copyright (2017), American Chemical Society.

The dataset for this study was compiled using a workflow that integrates the preferred reporting items for systematic reviews and meta‐analyses (PRISMA) [48] guidelines, NLP techniques, and manual curation, as illustrated in Figure 1a. The identification process involved searching the Scopus database with the keywords “nanoparticle,” “iron oxide,” “ferrite.” “superparamagnet*,” “magnet*,” “hyperthermia,” and “heat*,” yielding 19366 records. An NLP script was then employed during the screening step to analyze all records, selecting the top 1000 based on the frequency of relevant keywords in the titles and abstracts. These 1000 full‐text scientific articles underwent further examination using a second NLP script as part of the eligibility assessment phase.

During the eligibility phase, an initial analysis was conducted to identify predictive features based on the variables defined by the linear response theory (LRT) model and the experimental calorimetric SAR equation (Table S1; Equations 1–5) [7, 17, 49]. These features were comprised of eight intrinsic and extrinsic properties including the AMF amplitude (*H*) and frequency (ƒ), the concentration of SPION suspension (*C*_SPION_), and temperature for magnetic measurements (*T*). Due to the infrequent reporting of particle volume and magnetic susceptibility in literature, alternative parameters were selected, such as core diameter determined by transmission electron microscopy (*d*_TEM_), saturation magnetization (*M*_s_), coercivity (*H*_c_), and remanence (*M*_r_). Finally, the SAR was designated as the target feature. Figure 1b,c shows the results of the initial analysis. Figure 1b shows the distribution of articles reporting these eight features among the top 1000 screened articles. There was a notable disparity between the number of articles reporting the target feature, SAR (*n* = 357), and those reporting the predictive features, such as magnetic field (*n* = 894) and frequency (*n* = 481). Moreover, the incidence of articles discussing magnetic remanence (*n* = 43) was surprisingly low compared to those addressing other magnetic properties, such as coercivity (*n* = 327) and saturation magnetization (*n* = 514). These discrepancies were surprisingly unexpected, considering that experimental studies have shown that coercivity, remanence, and saturation magnetization are all related to SPION heating performance [33, 39, 50, 51]. Figure 1c shows a heatmap generated to visualize a sorted distribution of scientific articles based on how often they mention the target feature (SAR), along with all predictive features. This data visualization also showed a noticeable discrepancy between the number of scientific articles that reported SAR, concentration of SPION suspension, coercivity, and magnetic remanence. To address these discrepancies, a comprehensive manual review of the screened articles reporting SAR (*n* = 357) was conducted as part of the eligibility assessment. During this process, studies involving dopants such as nickel (Ni) [52, 53, 54] and gallium (Ga) [55, 56, 57] were excluded due to their limited relevance to biomedical applications, primarily owing to associated toxicological concerns [28]. Ultimately, the assessment identified 84 scientific articles that met the criteria for reporting all selected predictive features.

While the LRT model is widely used to estimate and benchmark the heating efficiency of SPIONs, it does not account for factors such as size distribution, surface coating, chemical composition, and particle aggregation. To address these limitations and enable a more comprehensive analysis, the evaluated set of nanoparticle properties and external parameters was expanded. As shown in Figure 1d, 22 additional predictive features were generated using information derived from the particle size distribution, core dimensional measurements, core shape, core chemical composition, field‐dependent magnetization curves (*M–H*), coating characteristics, and the suspension medium in which the hyperthermia measurements were performed. These additional features include standard deviation of the average core diameter (std*_d*_TEM_), estimated core volume (*V*_core_), estimated core surface area (*A*_core_), *A*_core_–*V*_core_ ratio, shape of the particle (spherical, cubic, octopod, octahedron, and nanoflower), dopant‐iron ratio (Mg─Fe, Mn─Fe, Co─Fe, and Zn─Fe), *M*_s_–*M*_r_ ratio, presence of nanoparticle coating, type of coating (multilayer composite, oil‐soluble, water‐soluble, inorganic), type of medium used for SPION suspension (non‐aqueous, low‐viscosity medium that allows nanoparticle movement, or medium containing stabilizers such as polymers or surfactants). The final dataset, comprising 1850 entries, was compiled by merging 18 numerical features including SAR and 13 categorical features.

A detailed overview of the categorization criteria used to describe coating materials, and the suspension medium is presented in Tables S2 and S3. Moreover, the distribution of all features, classified as numerical and categorical types, is also presented in Figure S1. It should be noted that these categorical features were encoded from qualitative information reported in the scientific literature. Therefore, they provide only a coarse‐grained representation of the underlying relationship between physical, chemical, and magnetic properties.

Most numerical features exhibited right‐skewed distributions, indicating that the majority of values are concentrated at the lower end of their respective ranges. The categorical features showed asymmetrical distributions, with certain categories being significantly more prevalent than others, such as spherical shape and organic coating. This suggests potential class imbalances that ultimately could lead to biased predictions towards the dominant categories.

A broad range of SAR values have been reported in the literature, from lower than 20 to higher than 2000 W g_Fe_^−1^ [22, 58]. This was also observed in our study, where SAR values from 0.1 to 2302 W g^−1^ [18, 59] were reported among the selected 84 scientific articles. The wide range of SAR of SPIONs arises from different compositions, sizes, shapes, size distributions, and the many different field strengths and frequencies that are often fixed by the configuration of the magnetic coils used to measure the SAR. Nonetheless, the SAR target feature showed a significant distribution of data points with values below 500 W g^−1^. Furthermore, the experimental conditions used to quantify the SAR, specifically the AMF amplitude (*H*) and frequency (ƒ), are predominantly below 500 Oe and 400 kHz, respectively. The distribution of SAR, *H*, and *f* values below specific thresholds likely reflects the adherence to the clinical tolerance limit of magnetic hyperthermia therapy [60, 61, 62, 63]. Additionally, the nanoparticle *M*_s_ values tend to cluster at 50–75 emu g^−1^ (Figure S1d), which is notably lower than the reported bulk magnetite range of 92–96 emu g^−1^ [64, 65]. In contrast, magnetic properties such as *M*_r_ and *H*_c_ predominantly exhibit values close to zero (Figure S1e,g), as expected for superparamagnetic materials. Similarly, the chemical features, expressed as the ratios of Zn─Fe, Co─Fe, Mn─Fe, and Mg─Fe, are highly skewed towards zero, indicating the use of low dopant levels in iron oxide nanoparticles.

The variation in physical, chemical, and magnetic properties of SPIONs, along with the differences in AMF parameters used for the determination of SAR, makes the comparison of data across studies highly challenging, particularly for traditional analytical methods. Additionally, inconsistencies in reporting SPION properties and SAR, which is either normalized by mass of SPION, Fe, or total metal, and the use of different measurement units further complicate the comparison. Therefore, the subsequent feature engineering process involved several critical steps, such as classifying categorical data, unifying units of measurement from numerical features, removing duplicates, transforming, normalizing, and scaling the data. These processing steps enabled the development of a machine learning framework capable of handling data heterogeneity, facilitating a more standardized analysis compared to conventional approaches.

Nevertheless, the intrinsic heterogeneity of the measurements reported in the literature might still introduce specific biases in the target SAR feature. For instance, SAR values have been reported as difficult to compare due to differences in magnetic properties and experimental conditions [66]. Moreover, variations in measurement techniques and fitting procedures can introduce systematic errors that may persist after unit normalization [12, 67], leading to residual biases that cannot be entirely removed from the ML predictive model. Consequently, the performance of the reported model should be interpreted acknowledging those limitations.

### 2.2 Data Splitting Strategy, Normalization, and Exploratory Analysis

The dataset was divided into four subsets (training, validation, calibration, and test) using a stratified sampling approach. Initially, categorical labels representing the characteristics of shape, doping, and coating were generated and combined into a unified stratified label. The dataset was then partitioned using this stratified label to maintain equally distributed label proportions across all subsets, as detailed in Figure S2a. A complete description of the stratified labels and their respective weights is displayed in Table S4. Specifically, 70% of the data was sampled and assigned as the training subset, while the remaining data points were equally distributed among the validation, calibration, and test subsets. These four subsets were subsequently utilized for model development, hyperparameter tuning, uncertainty quantification, and final model performance evaluation, respectively. The data distribution of each feature after transformation and scaling is detailed in Figure S2b. Notably, the categorical features reported a consistent class balance, while continuous features showed similar shape patterns across all subsets.

Subsequently, a comparative analysis utilizing both a principal component analysis (PCA) and t‐distributed stochastic neighbor embedding (t‐SNE) was conducted to visualize the linear and nonlinear relationships between the predictive features. The results of these analyses are shown in Figure S3. The PCA plot showed the data distribution of all subsets along the first two principal components, which explains 25.5% and 15.6% of the variance, respectively. The projected data points on the two principal components space showed some grouping patterns with a few tight clusters. However, the majority of data points were scattered, and the transitions between clusters were not well defined. These results suggest that PCA has effectively captured the primary linear relationships between features but may have overlooked more complex nonlinear feature relationships. In contrast, the t‐SNE plot revealed a data distribution characterized by smaller, well‐defined, and better‐dispersed clusters in the area defined by the two t‐SNE principal components. Given the notable ability of t‐SNE to capture complex nonlinear interactions between features [69], the comparison between PCA and t‐SNE plots suggested the presence of secondary nonlinear relationships within the predictive features. Furthermore, the projected data points obtained from the training, validation, calibration, and test subsets were intermixed and clearly identified in all clusters reported in the t‐SNE analyses. This result indicated that the predictive features exhibited a similar data distribution across all subsets, and the data‐splitting process did not introduce any significant bias.

### 2.3 Model Building Based on Bayesian Hyperparameter Optimization

Bayesian optimization is a sequential model‐based method applied to solve optimization problems [70]. This method efficiently finds hyperparameter values that can significantly improve the performance of ML models [71]. It uses an approximation technique to predict the performance of various hyperparameter configurations based on the results of previous model evaluations [70, 72]. In this study the Bayesian optimization process iterated 1000 times to search for the optimal hyperparameter values within a predefined search space, as detailed in Table S5. The hyperparameter optimization was focused on reducing the weighted root mean squared error (wRMSE) on the validation subset and not introducing excessive model variance. The risk of overfitting during the optimization process was controlled by monitoring the difference in training and validation wRMSE metrics across the 1000 study iterations. After the study, the output trials were filtered to those within the top 10% based on validation wRMSE and subsequently ranked by the smallest training‐validation gap, selecting the trial with the lowest overall gap. This gap‐based selection strategy directly penalizes model hyperparameters that fit the training data disproportionally well, thereby reducing the likelihood of overfitting to the training subset.

Twelve ML algorithms were explored in this stage, including lasso regression (LR), ridge regression (RR), elastic net regression (ENR), partial least squares (PLS), support vector regressor (SVR), k‐nearest neighbors (KNN), decision tree regressor (DTR), random forest regressor (RFR), categorical boosting regressor (CatBoost), light gradient boosting (LightGBM), extreme gradient boosting (XGBoost), and deep neural network (DNN). These algorithms were selected to cover a broad range of predictive methodologies, including linear, nonlinear, tree‐based, boosting‐based, and deep learning. This selection is consistent with similar ML‐based studies focused on optimizing and developing materials for applications in life sciences [42, 46, 73, 74]. The overall wRMSE reduction for the 12 ML models evaluated in this study is illustrated in Figure S4. Notably, the lasso regression, ridge regression, elastic net regression, partial least squares, and k‐nearest neighbors models did not exhibit a significant wRMSE reduction over the 1000 iterations. Moreover, the decision tree regressor and random forest regressor models achieved moderate wRMSE reduction but still reported higher values compared to the support vector regressor, boosting‐based, and DNN models. Boosting‐based models, including CatBoost, LightGBM, and XGBoost, demonstrated a significant reduction in wRMSE metrics obtained from the validation and training subsets. The optimal hyperparameter values for each model are also summarized in Table S5.

Figures S5–S8 detail the top five most significant hyperparameters in terms of wRMSE reduction for each model. The hyperparameter search space was adjusted to minimize the wRMSE of the validation subset while reducing the gap between wRMSE values calculated from the validation and training subsets. This approach facilitates the selection of the optimal hyperparameter values for each model, ensuring an appropriate balance between bias and variance.

The optimization of the SVR model demonstrated that using nonlinear kernels resulted in a reduced wRMSE, whereas the linear kernel tended to increase the error in model predictions (Figure S5). Among the tree‐based models, the optimization of the DTR model revealed that increasing the number of features and leaf nodes was associated with lower wRMSE (Figure S6a). Similarly, the optimization of the RFR model demonstrated that an increase in the number of nodes in each decision tree resulted in a lower wRMSE. In contrast, a higher wRMSE was correlated with an increase in the number of samples required for a node, as is detailed in Figure S6b.

Regarding boosting‐based models, the optimization of the CatBoost model revealed that deeper trees and a higher number of iterations were linked to a lower wRMSE value (Figure S7a). Similarly, the LightGBM model optimization revealed that deeper trees and a higher number of leaves in a tree were associated with lower wRMSE (Figure S7b). Lastly, the XGBoost model optimization showed a similar pattern, wherein deeper trees and a higher number of estimators were linked to a lower wRMSE (Figure S7c). For the CatBoost, LightGBM, and XGBoost models, an early‐stopping callback was used to terminate boosting when the validation wRMSE stopped improving, therefore avoiding unnecessary increases in model complexity. These observations are consistent with recent studies using ML to predict the physicochemical properties of nanoparticles for biomedical applications [75, 76]. Overall, these findings suggest that tuning the tree depth, number of estimators, and iterations leads to lower error metrics and improves the model performance in this domain.

The DNN model optimization showed that increasing the batch size was related to a reduction of the wRMSE value, whereas changes in the dropout rate and the regularization parameters did not generate any visible improvement in the model predictions (Figure S8a). Furthermore, the design of the neural network architecture was assessed by applying the same Bayesian optimization approach (Figure S8b). This approach suggested that the optimal architecture identified consists of three deep layers containing 239, 242, and 88 neurons, respectively. For the DNN model, a specific number of epochs was selected from the learning‐curve plateau region to prevent the increment of model complexity. Among all the evaluated epoch numbers, the DNN model trained for 450 epochs demonstrated superior performance metrics (Table S10).

### 2.4 Model Comparative Box‐Plot Analysis

**FIGURE 2:**
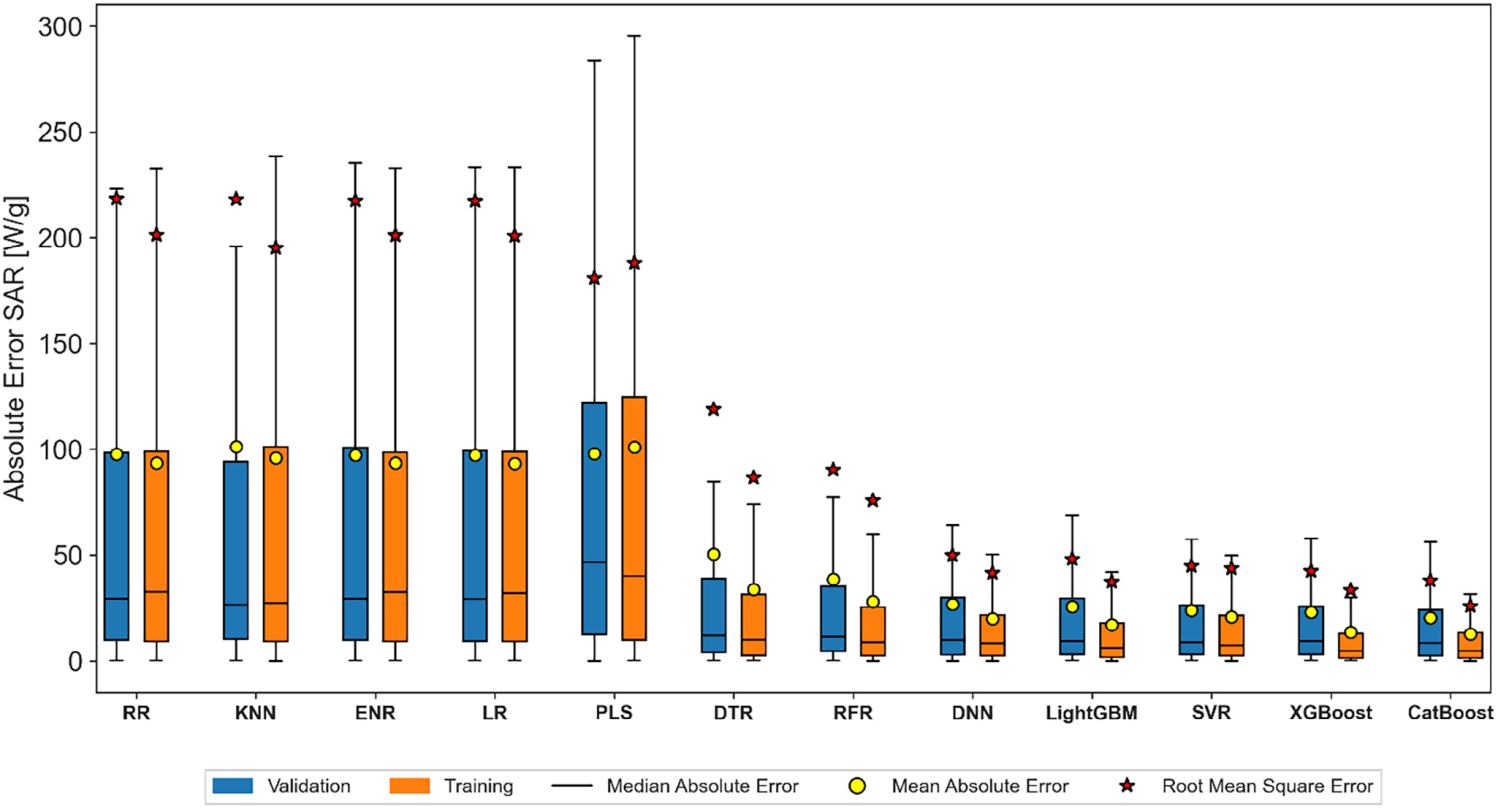
Overall predictive performance after hyperparameter tuning. The boxplots detail the absolute error dispersion in the SAR predictions (W g^−1^) from validation and training subsets. Each boxplot illustrates the median absolute error (MedAE; black lines), mean absolute error (MAE; yellow circles), and root‐mean‐squared error (RMSE; red stars).

Following the selection of the optimal hyperparameters, a comparative box‐plot analysis of the absolute error was conducted, as illustrated in Figure 2 and detailed in Table S6. This analysis aimed to identify the model achieving both the lowest mean absolute error (MAE) and root mean squared error (RMSE) on the validation subset, and the narrowest distribution of absolute error, quantified by its standard deviation (AE_std_). The results indicated that linear models, such as LR, RR, ENR, and PLS, exhibited wider AE_std_ distributions with values ranging from 195 to 152 (SAR, W g^−1^). This tendency was further supported by their relatively higher MAE and RMSE metrics, which reported values as high as 101 and 218 (SAR, W g^−1^), respectively.

In contrast, nonlinear models showed lower MAE and RMSE metrics along with generally narrower AE_std_, indicating superior performance and more consistent predictions. Among these models, DTR achieved validation MAE and RMSE values of around 51 and 119 (SAR, W g^−1^), respectively, while the RFR model showed enhanced performance with MAE and RMSE values of 39 and 90 (SAR, W g^−1^), respectively. The DNN model showed improved performance, with MAE and RMSE values just above 27 and 50 (SAR, W g^−1^). Notably, the SVR model achieved similar performance with validation MAE and RMSE metrics of 24 and 45 (SAR, W g^−1^), respectively.

Boosting‐based models exhibited even higher performance, with LightGBM achieving validation MAE and RMSE values around 26 and 48 (SAR, W g^−1^), respectively. Moreover, the XGBoost model showed slightly improved metrics, with validation MAE and RMSE values above 23 and 42 (SAR, W g^−1^), respectively. Lastly, the CatBoost model showed the lowest validation MAE and RMSE values among all evaluated models, achieving values around 20 and 38 (SAR, W g^−1^), respectively. This model also showed the narrowest validation AE_std_ of 32 (SAR, W g^−1^).

**FIGURE 3:**
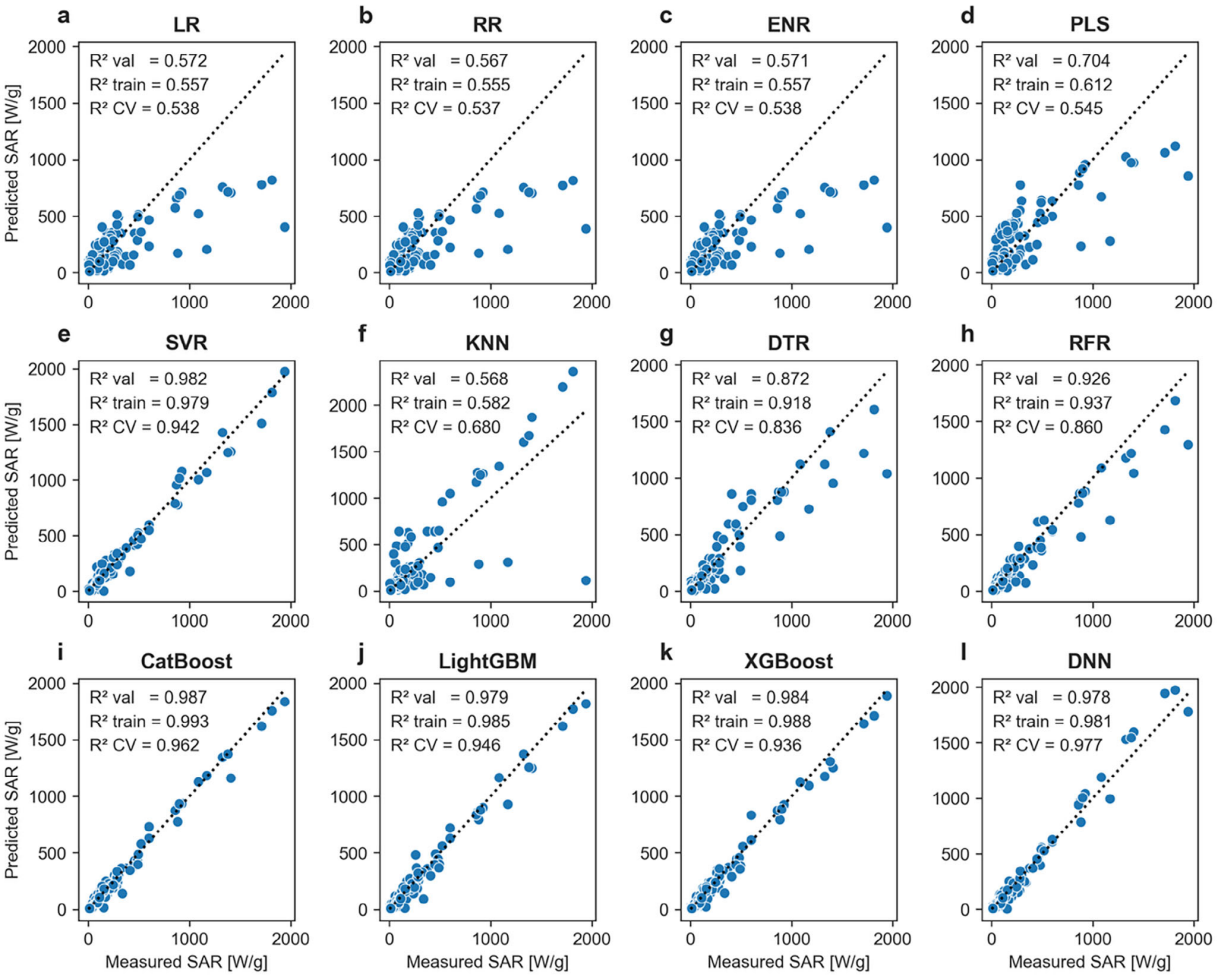
Comparison of regression models based on the coefficient of determination (*R* ^2^). In each subpanel, blue dots represent the predicted specific absorption rate (SAR) from the validation subset versus the experimentally measured values. *R* ^2^ fivefold cross‐validation scores from the training subset are represented by CV. Panels (a) through (d) illustrate the performance of linear models, while panels (e) through (l) show the performance of nonlinear models.

Furthermore, a comparative analysis of the coefficient of determination (*R* ^2^) was conducted to assess how well each model fits the data (Figure 3). The evaluated linear models, including LR, RR, and ENR, demonstrated poor predictive performance, showing low *R* ^2^ values hovering around 0.5 to 0.6 for both training and validation subsets. The wide dispersion of data points around the identity line further indicated significant discrepancies between the predicted and measured values, highlighting the limitations of linear models in capturing the complex relationships within the predictive features that affect SAR. The KNN and PLS models were excluded from further analysis due to noticeable inconsistencies between their reported metrics, where the validation metrics outperformed the training *R* ^2^, RMSE, and MAE.

In contrast, nonlinear models, such as SVR, DTR, and RFR, exhibited a notably improved performance. Specifically, the DTR and RFR models achieved *R* ^2^ values above 0.9 for the validation and training subsets, indicating improved predictive performance and generalization capabilities. The clustering of data points around the identity line for these models suggests a better model fit and highlights the benefits of nonlinear regression models in SAR predictions. Moreover, the SVR and boosting‐based models, including CatBoost, LightGBM, and XGBoost, as well as the DNN model, emerged as the top five models with the best performances. These models achieved *R* ^2^ values exceeding 0.97 for both validation and training subsets, with XGBoost and CatBoost showing particularly high predictive performance. Moreover, the predicted data points from these models closely aligned with the identity line, reflecting minimal discrepancies between the predicted and measured SAR values. The agreement between predicted and measured SAR values highlights the robustness of the nonlinear models in capturing complex relationships between nanoparticle properties and SAR.

### 2.5 Model Agglomerative Hierarchical Clustering Analysis

SVR, CatBoost, LightGBM, XGBoost, and DNN were identified as the five top‐performing models in predicting SAR after training with 30 predictive features. However, using an excessive number of features might increase the risk of introducing multicollinear correlations between them, thereby affecting the model stability and interpretability [77, 78]. To address this potential issue, these models were evaluated through an agglomerative hierarchical clustering analysis to identify potentially redundant predictive features.

**FIGURE 4:**
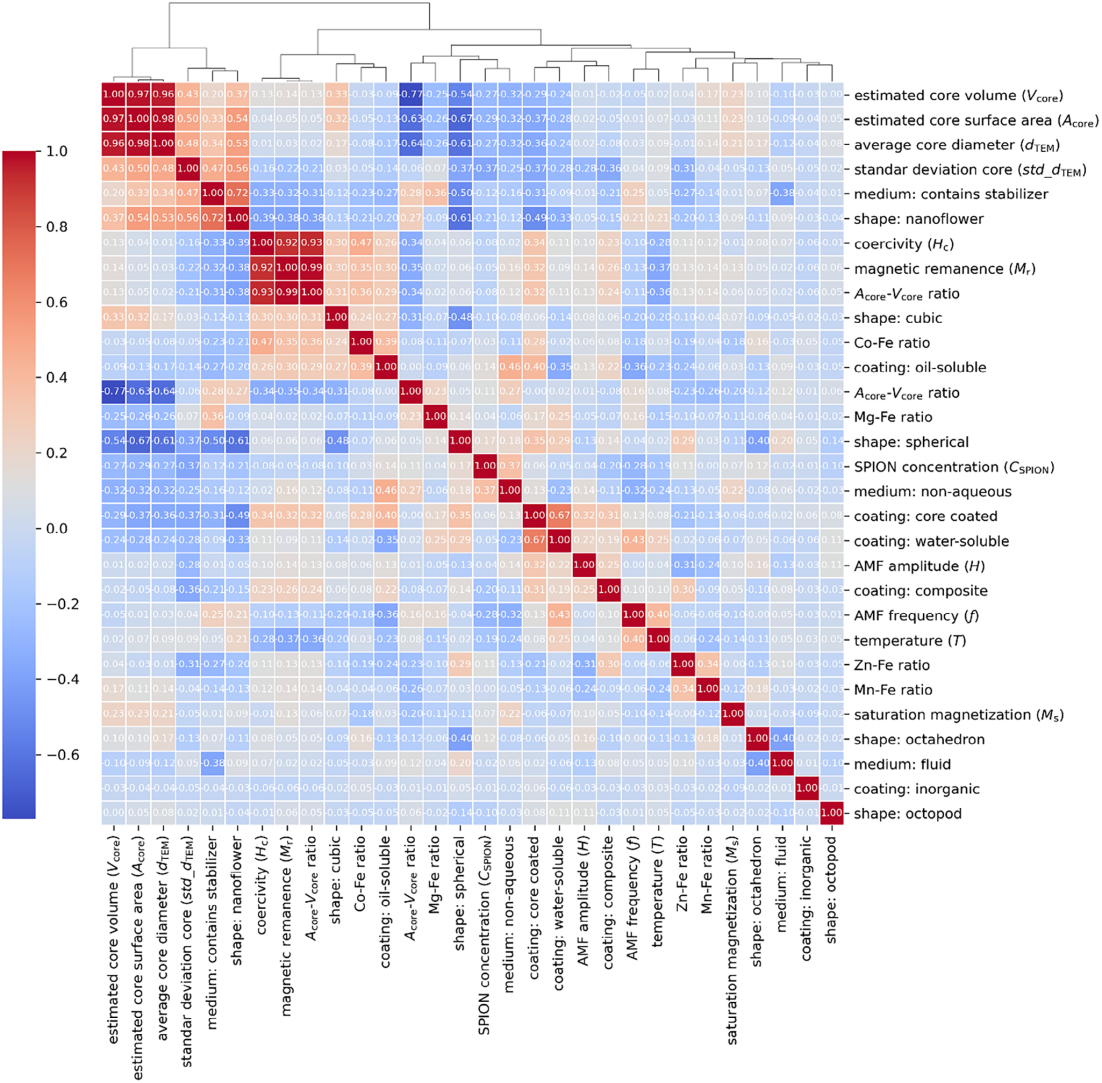
Agglomerative hierarchical clustering analysis of predictive features. Cells colored red represent a Spearman correlation coefficient of 1. As the correlation coefficient decreases, the intensity of the red color fades, indicating lower values until it reaches 0. Negative values of the correlation coefficient are shown in blue‐colored cells. The intensity of the blue color increases as the values range from 0 to −0.8. The dendrogram at the top shows the hierarchy of the 30 included features.

A heatmap displaying the Spearman correlation coefficients (*ρ*) between all the features was created (Figure 4). The minimum variance method was then applied for hierarchical clustering, revealing four clusters of highly correlated features that surpassed the 0.7 threshold [77]. The first cluster revealed that physical properties such as estimated core volume (*V*_core_), estimated core surface area (*A*_core_), and average core diameter (*d*_TEM_) presented the highest correlation coefficients (*ρ ≥* 0.96). The second cluster highlighted a strong negative correlation between the *A*_core_–*V*_core_ ratio and *V*_core_ (*ρ* = −0.77). Together, these two clusters confirmed significant interdependence among core dimensional features derived from *d*_TEM_. The third cluster showed strong correlations between magnetic properties such as coercivity (*H*_c_), magnetic remanence (*M*_r_), and the *M*_r_–*M*_s_ ratio (*ρ*: 0.91–0.98). This agrees with our previous experimental study that reported a high correlation coefficient (*r* = 0.99) between *M*_r_ and *H*_c_ [39]. Lastly, the fourth cluster detected moderate correlations (*ρ* = 0.72) between nanoflower‐shaped cores and the categorical feature that describes the stabilizer‐containing medium for suspending nanoparticles.

**FIGURE 5:**
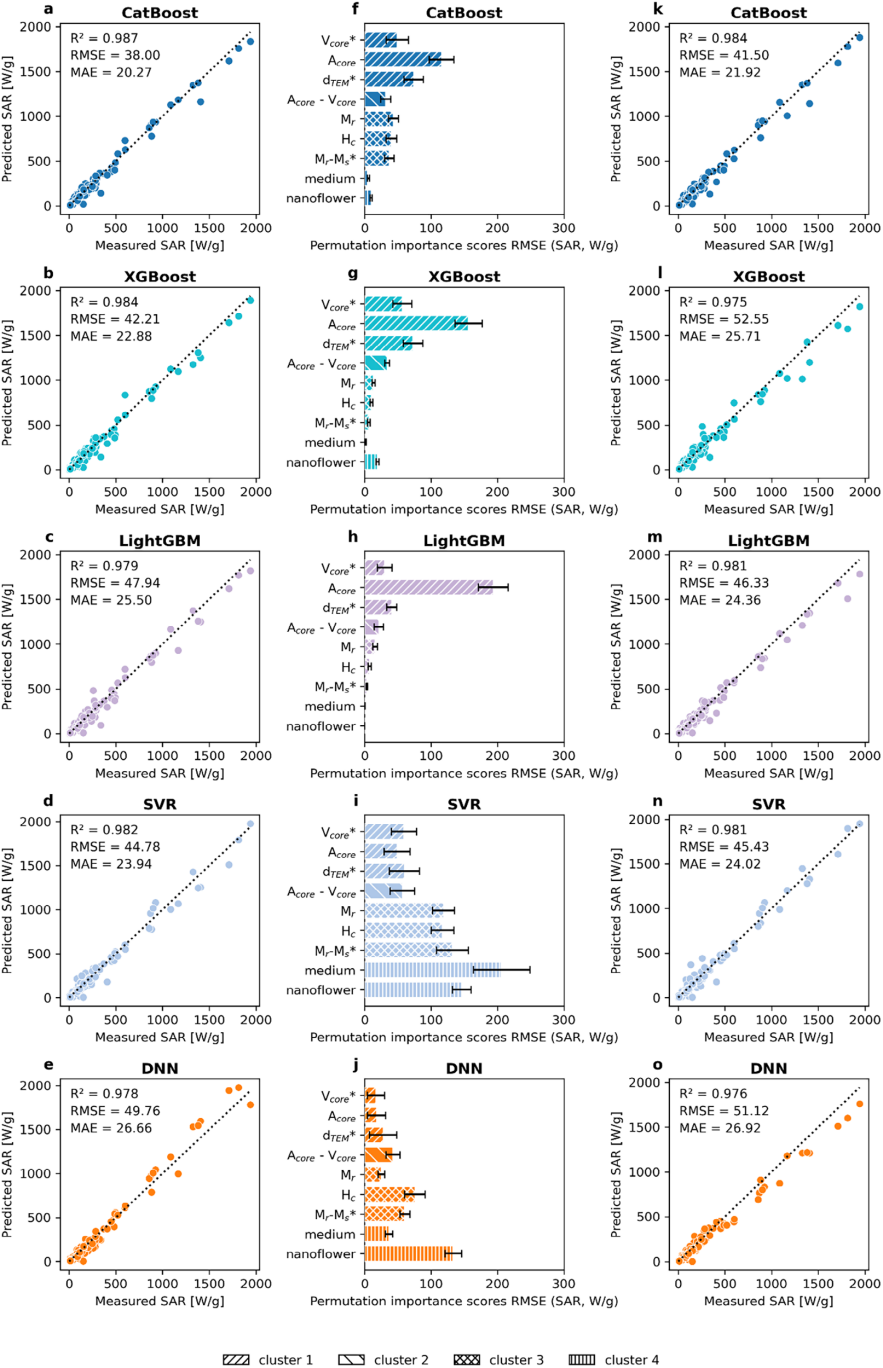
Overall model performance using the CatBoost, XGBoost, LightGBM, SVR, and DNN models. (a–e) Initial model performance with 30 features. (f–j) Feature importance analysis of highly correlated features clustered in three groups. The features selected to be removed are marked with an asterisk (*). Error bars were generated after ten iterations of random shuffling of the values of each feature. The feature importance scores were calculated as the RMSE (SAR, W g^−1^). (k–o) Model performance after removal of the *V*_core_, *d*_TEM_, and *M*_s_–*M*_r_ ratio features.

Initially, the five top‐performing models were trained using all 30 predictive features, some of which exhibited high positive and negative correlation coefficients (*ρ* ≥ 0.7 and *ρ* ≤ −0.7). The initial performance metrics are reported in Figure 5a–e. Subsequently, a permutation importance analysis was conducted to evaluate the relative importance of each highly correlated feature within each cluster (Figure 5f,g). The nature of each feature was also assessed to determine whether it represented an individual property (*d*_TEM_, *M*_r_) or was derived from a combination of properties (*A*_core_–*V*_core_ ratio, *M*_r_–*M*_s_ ratio).

The analysis reported a general pattern across the permutation importance rankings. All the evaluated boosting‐based models, such as CatBoost, XGBoost, and LightGBM, reported *A*_core_ as the most important physical feature, while magnetic features and categorical features such as “shape: nanoflower” and “medium: contains stabilizer” displayed permutation importances that were four‐fold to ten‐fold lower. On the contrary, both the DNN and SVR models assigned higher permutation importance scores to magnetic features such as *M*_r_, *H*_c_, and *M*_r_–*M*_s_ ratio. The scores of these features were two‐fold to four‐fold higher than those reported from physical features. Moreover, categorical features reported even higher scores in both models. Based on this analysis, the features estimated core volume (*V*_core_), average core diameter (*d*_TEM_), and *M*_r_–*M*_s_ ratio were removed to evaluate their impact on the overall model performance. The models were then retrained without these features, and their performance was assessed using the validation subset (Figure 5k–o).

The retrained models generally exhibited an increase in MAE and RMSE error metrics. Specifically, the CatBoost and XGBoost models showed an increase in MAE from 20 to 22 and from 23 to 26 (SAR, W g^−1^), respectively. A similar pattern was observed in RMSE metrics from 38 to 42 and from 42 to 53, respectively. Moreover, models such as SVR and DNN showed a negligible increase of approximately 1 unit in both MAE and RMSE metrics. In contrast, the LightGBM model showed a slight decrease in MAE from around 25 to 24 (SAR, W g^−1^) and in RMSE from above 48 to 46 (SAR, W g^−1^). Furthermore, the *R* ^2^ metrics exhibited only negligible changes following the feature reduction. All five evaluated models maintained high *R* ^2^ values ranging between 0.97 and 0.98. Overall, these findings suggest that the removal of highly correlated features with lower permutation importance scores had a minimal impact on the overall model performance. The slight variations observed in the performance metrics indicate that the inclusion of highly correlated features is unlikely to affect model performance due to multicollinearity. On the contrary, the removal of these features reduced the model performance in most of the cases. Therefore, the five top‐performing models trained with 30 features were considered for further evaluation.

### 2.6 Model Conformal Prediction Analysis

**FIGURE 6:**
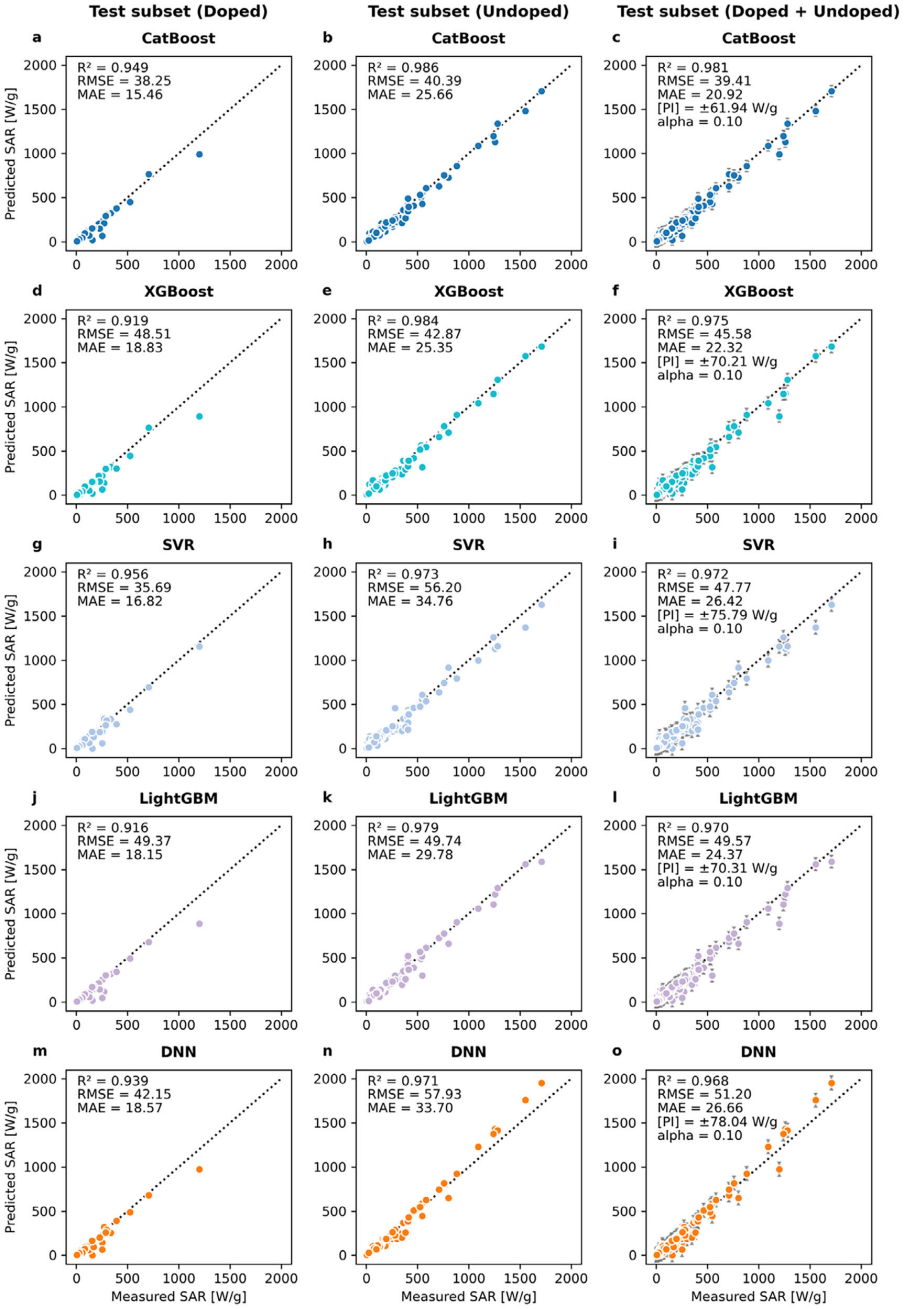
Model prediction performance on the test subset. Panels (a) through (o) show the results of CatBoost, XGBoost, SVR, LightGBM, and DNN models, respectively. Performance metrics (*R* ^2^, RMSE, MAE) are reported separately for doped, undoped, and combined nanoparticles across all evaluated models. The x‐axis displays the measured specific absorption rate (SAR) values, while the y‐axis displays the predicted SAR values. Prediction intervals (PI) are represented by the gray error bars, while the black dotted line indicates the identity line.

The five top‐performing models were evaluated through conformal prediction analysis using the calibration and test subsets. Conformal prediction is a statistical methodology that enhances the reliability of ML models by generating prediction intervals that are guaranteed to contain the true outcome with a specified confidence level [79]. This technique involves computing a nonconformity measure to quantify the extent to which a new example diverges from the observed data, enabling the construction of prediction intervals or regions likely to contain the true outcome with a specified confidence level [80, 81]. Figure 6 presents a comparative analysis of the prediction performance of these models, each utilizing 30 features. It also illustrates the prediction interval (PI) generated by the conformal prediction technique with a 90% coverage probability (*α* = 0.10). The DNN, SVR, LightGBM, and XGBoost models demonstrated strong predictive capabilities with *R* ^2^ values > 0.97 on the test subset and PI around ± 78, 76, 70, and 70 (SAR, W g^−1^), respectively (Figure 6b,e). Overall, the CatBoost model demonstrated superior performance with the highest *R^2^* value of 0.98 on the test subset (Figure 6a), indicating the best fit among the top‐performing models. This model was then selected for further analysis because it achieved the lowest error metrics, with MAE and RMSE values of 21 and 39 (SAR, W g^−1^), respectively, and the narrowest PI of ± 62 W g^−1^ for an expected output range of 0‐2000 (SAR, W g^−1^), indicating the highest predictive performance.

The width of the PI corresponding to the CatBoost model was then assessed across four expected output SAR ranges, as detailed in Figure S9. A noticeable trend was identified among all the evaluated ranges. The initial PI of ± 62 decreased to ± 51, 48, 40, and 27 W g^−1^ for the expected ranges of 0–1000, 0–500, 0–250, and 0–100 (SAR, W g^−1^), respectively. These expected ranges are particularly relevant when considering the batch‐to‐batch variability commonly seen in experimental SAR measurements. For instance, discrepancies as high as Δ39 W g^−1^ have been reported in the 0–100 W g^−1^ range for spherical nanoparticles [21], whereas cubic nanoparticles in the 0–250 W g^−1^ range have reported differences up to Δ143 W g^−1^ [82]. Furthermore, individual measurements in the 0–500 W g^−1^ range have shown variations as high as Δ200 W g^−1^, while an experimental error of ≈5% has been reported for SAR values in the 0–1000 W g^−1^ range [12, 83]. Notably, for all evaluated ranges, the conformal prediction intervals derived from the CatBoost model remain consistently narrower than the experimental error margins reported in the literature. These experimental error margins included sources of variability that were not included in the CatBoost model, such as long‐term stability [21], and batch‐to‐batch differences in SPION crystallinity [12, 83].

### 2.7 Quantifying Factors Influencing Magnetic Hyperthermia

In data‐driven studies of complex phenomena such as magnetic hyperthermia, interpretability is essential for elucidating the underlying relationships between predictive features and the subsequent practical implementation of the findings [84]. Therefore, a feature importance analysis was performed to understand the contribution of these features to the CatBoost model. Moreover, the Shapley additive explanation (SHAP) method was applied to quantify the contribution of all 30 predictive features.

**FIGURE 7:**
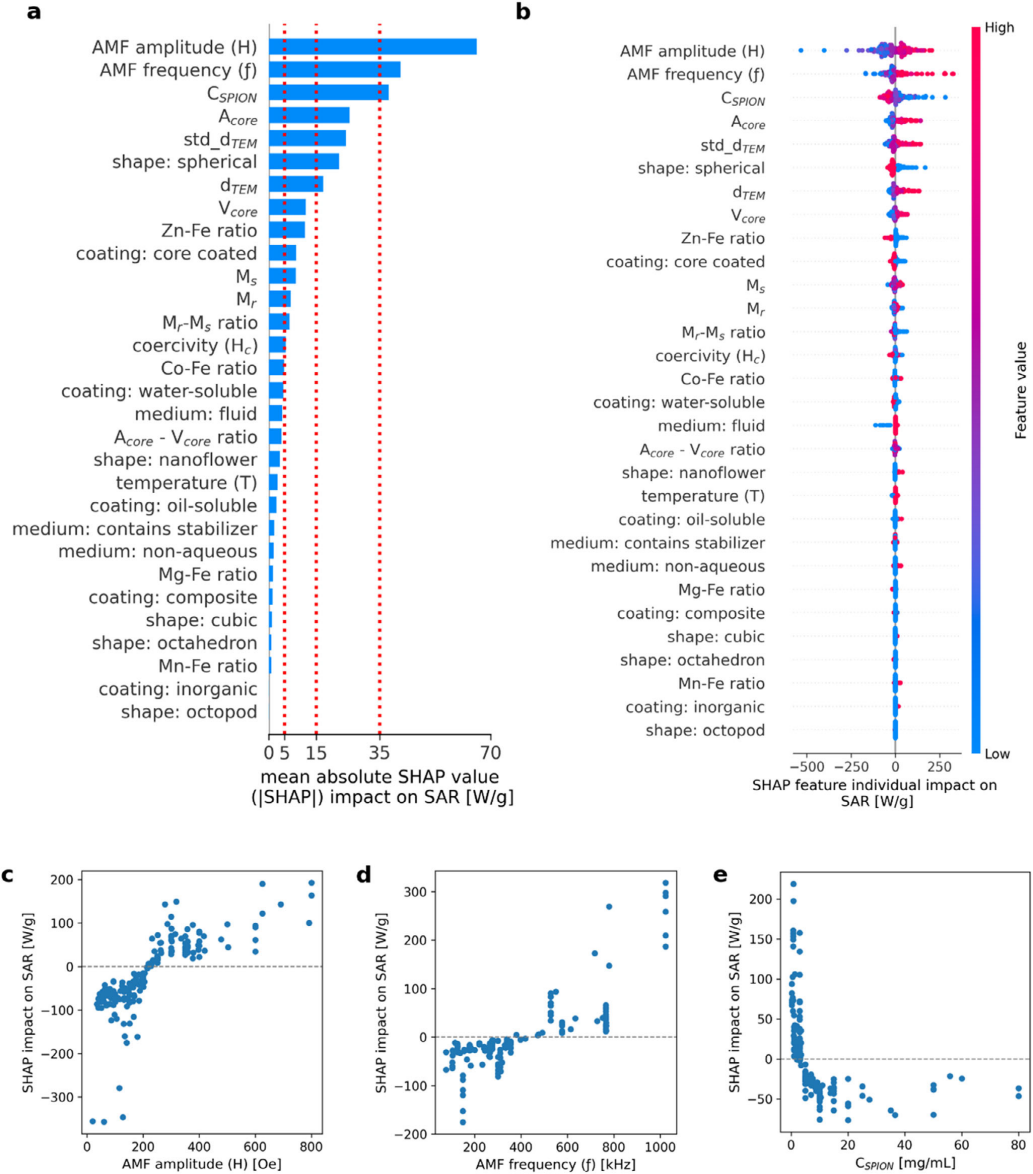
(a) Shapley additive explanations (SHAP) plot showing the mean absolute SHAP values of all 31 features evaluated by the CatBoost model. (b) Shapley additive explanations (SHAP) summary plot for the CatBoost model on the test subset. The feature importance of the SAR prediction is displayed in the y‐axis from higher to lower importance. The red‐to‐blue‐colored dots on the x‐axis represent the positive or negative contribution of each feature on each instance in the model predictions. Red colored dots represent high feature values for a particular instance. Blue colored dots represent low feature values. (c–e) SHAP dependence plots of factors with highest impact on SAR, viz., AMF amplitude (c), frequency (d), and *C*_SPION_ (e).

The mean absolute SHAP value of all features is presented in the SHAP global bar plot (Figure 7a), offering a measure of feature importance across the entire dataset. The predictive features were categorized as considerable (>10%, >35 W g^−1^), moderate (5%–10%, 15–35 W g^−1^), weak (1.5%–5%, 5–15 W g^−1^), and negligible (<1.5%, <5 W g^−1^) based on their averaged contribution to the SAR (W g^−1^) value. Moreover, a detailed representation of the impact of each feature based on individual samples is also presented in the SHAP beeswarm summary plot (Figure 7b).

Overall, the extrinsic experimental features reported a considerable impact on SAR, with amplitude (*H*) and frequency (ƒ) being the most influential (Figure 7a,b). High values of both features were associated with increased SAR, which is consistent with the LRT, and previously published theoretical and experimental studies [85, 86, 87]. The SHAP dependence plot of AMF amplitude shows a nonlinear relationship, where SAR increases steeply with amplitude at lower magnetic fields, in agreement with LRT, but plateaus at higher AMF amplitudes (Figure 7c). This deviation occurs because LRT assumes a linear magnetic response, valid only when the field is weak compared to thermal energy [49]. However, this approximation breaks down at higher magnetic field values as the nanoparticles approach magnetic saturation, and hysteresis losses dominate the heating, particularly from particles with high anisotropy or larger size within the SPION sample [88]. The positive correlation of AMF frequency with SAR is also consistent with LRT (Figure 7d). However, the relationship is not strictly linear across the entire range. At low frequencies, SHAP values fluctuate near zero, suggesting a limited contribution to SAR, while a clear increase is observed only beyond ≈400 kHz. This behavior likely reflects the frequency dependence of magnetic relaxation processes, particularly Néel relaxation, which becomes more efficient when the excitation frequency (*f*) approaches the inverse of the characteristic relaxation time (*τ*_N_) of the nanoparticles (Table S1; Equations 3 and 4). It is important to consider that high AMF frequency and amplitude can trigger adverse biological effects, such as nerve stimulation or eddy current‐induced tissue heating [89]. To avoid such risks, safety guidelines recommend limiting the *f* x *H* product under 4.85 × 10^8^ A m^−1^ s^−1^, with considerations given to the type of tissue and the geometry of the magnetic coil [60, 62, 63, 89].

The concentration of SPIONs (*C*_SPION_) also showed a considerable impact on the prediction of hyperthermia performance, with low values associated with an increase in SAR (Figure 7e). Increasing particle concentration initially leads to reduced heating efficiency, likely due to enhanced magnetic dipole–dipole interactions and agglomeration effects, which can suppress effective relaxation mechanisms and limit SAR. Additionally, a significant increase in SPION concentration can alter the overall specific heat capacity and viscosity of the suspension, decreasing the SAR (Table S1 and Equations 1 and 5). This finding aligns with previous research that reported a correlation between decreasing SAR values and increasing SPION concentration [90, 91, 92, 93]. At high concentrations (>10 mg mL^−1^), SHAP values stabilize, indicating a saturation point where additional SPIONs no longer significantly alter SAR due to already‐maximal interparticle interactions and suppressed relaxation dynamics.

The estimated surface core area, the standard deviation of core diameter (std*_d*_TEM_), spherical shape, and the average core diameter (*d*_TEM_) were categorized as moderately impactful features. Magnetic relaxation processes (particularly Néel relaxation) are exponentially sensitive to the particle size, with optimal SAR occurring within a narrow diameter window (Table S1 and Equations (1), (2), (3)). Slight deviations from this optimal size can drastically alter relaxation times and thus heating efficiency. Literature reports frequently demonstrate bell‐shaped or size‐dependent SAR trends, with optimal diameters typically in the range of 12–20 nm for SPIONs under clinical AMF conditions [94]. While core diameter is a widely reported and intuitive size descriptor, it scales linearly and may not fully capture the functional effect of the full particle size on magnetic heating efficiency. In contrast, core area (∝ diameter^2^) more accurately reflects the magnetic cross‐section that interacts with the applied alternating magnetic field, making it more sensitive to changes in SAR. This is reflected in the SHAP plot (Figure 7a,b), where the core surface area showed a stronger impact on SAR prediction. Interestingly, the increase in the standard deviation of the average core diameter showed a positive correlation with SAR. This trend might be supported by the increased probability of optimal‐sized nanoparticle subpopulations in more polydisperse samples. However, the non‐normalized nature of the standard deviation metric may accentuate this effect. Future SAR predictive models could explore normalized SPION size descriptors such as the coefficient of variation or relative standard deviation.

It is important to highlight that core volume rather than core diameter is one of the key factors affecting the SAR of SPION nanoparticles (Table S1 and Equations (1), (2), (3), (4)). Particles of the same diameter but different shapes can vary in calculated volumes and area [95, 96]. However, our model showed a weaker impact of volume on SAR compared to both core area and core diameter.

The performance of nanoparticles in magnetic hyperthermia is shape‐dependent. While simple geometries can be described using basic dimensions, complex shapes such as nanoflowers cannot be accurately described by diameter or area alone. The spherical shape exhibited the highest influence among all shape features, where low values of this feature, which indicate non‐spherical shapes, were linked to a moderate increment in the predicted SAR values. Although other shapes influenced the SAR prediction weakly or negligibly, their high values were related to an increase in SAR, in agreement with the literature [23, 97]. Due to limited sample availability, current models struggle to capture geometric complexity. Advanced data representations, such as transmission electron microscopy images, could offer a more accurate alternative. As larger and more diverse datasets become available, image‐based learning will play a pivotal role in advancing SPION design for hyperthermia applications.

From a mechanistic perspective, the prominence of amplitude, frequency, and size‐related descriptors in the SHAP analysis reflects their collective influence on the Néel relaxation time (τN), which depends exponentially on effective anisotropy energy and magnetic volume. However, as effective anisotropy constants and energy barrier distributions are rarely reported across the literature, experimentally reported parameters such as H, f, and particle dimensions serve as proxies for the underlying relaxation dynamics. Therefore, the resulting SHAP interpretation remains necessarily phenomenological, while still consistent with the established mechanistic framework governing magnetic hyperthermia.

Among the SPION composition features, the Zn─Fe ratio was identified as the most impactful chemical feature, with high values leading to a marginal decrease in SAR. Experimental evidence supports this observation, indicating that when the doping fraction *x* exceeds 0.1 in Zn_x_Fe_3−x_O_4_ nanoparticles, the resulting SAR value decreases [35, 37]. In contrast, high feature values in the Co─Fe ratio were associated with a marginal increment in the SAR prediction output. Similarly, this finding is supported by experimental results that reported a positive correlation between SAR and the doping fraction *x* in Co_x_Fe_3−x_O_4_ nanoparticles [15, 51]. Further, the Mg─Fe and Mn─Fe ratios had a negligible influence on the model output.

Saturation magnetization showed a weak impact on the overall SAR prediction. Nonetheless, it was identified as the most influential magnetic property, where high values were linked to a marginal increase in the model output. This trend is consistent with experimental results, which have reported a correlation between an increase in *M*_s_ and approximately three‐ to five‐fold increase in SAR values [33, 47, 98]. Although, magnetic remanence and coercivity presented mixed results, low values of coercivity were linked to an increase in SAR, consistent with our previous experimental results [39]. The *M*_s_–*M*_r_ ratio displayed a discernible pattern, where low feature values correlated with a marginal increase in the SAR output. These findings suggest that magnetic anisotropy, a key factor influencing both coercivity and relaxation mechanisms, may play a critical role in governing SAR, even though it was not explicitly accounted for in the current model due to limited reporting across the literature.

The coating‐related features exhibited negligible influence on the model output, showing no noticeable differences between coating categories such as oil‐soluble, water‐soluble, inorganic, or multi‐layer composite coatings. The only discernible trend was observed in the core coating characteristics, where uncoated cores unexpectedly reported a marginal increase in the SAR output. This trend can be correlated to the non‐aqueous feature values, suggesting that the test subset included uncoated nanoparticles measured in a non‐aqueous medium, which resulted in high SAR values.

Lastly, the temperature at which magnetic properties of SPIONs were measured is often reported in literature but rarely correlated to hyperthermia performance. Our study indicates that this feature has a negligible impact on the prediction of SAR.

### 2.8 Model Performance and Limitations

The performance of the CatBoost model was evaluated by predicting the hyperthermia performance of an independent dataset derived from two recently published studies [39, 99] that were not included in any of the previously defined subsets. This new dataset included 12 nanoparticle samples, each characterized by a *d*_TEM_ ranging from ≈7 to ≈30 nm and chemically doped with Zn, Mn, Mg, and Co. All the samples were coated, with the coating composition varying based on the dopant. The Zn‐, Mn‐, and Mg‐doped nanoparticles were coated with citrate, while Co‐doped nanoparticles were coated with a mixture of oleic and polyacrylic acid. A detailed description of the physicochemical and magnetic properties of these samples is provided in Table S7.

**FIGURE 8:**
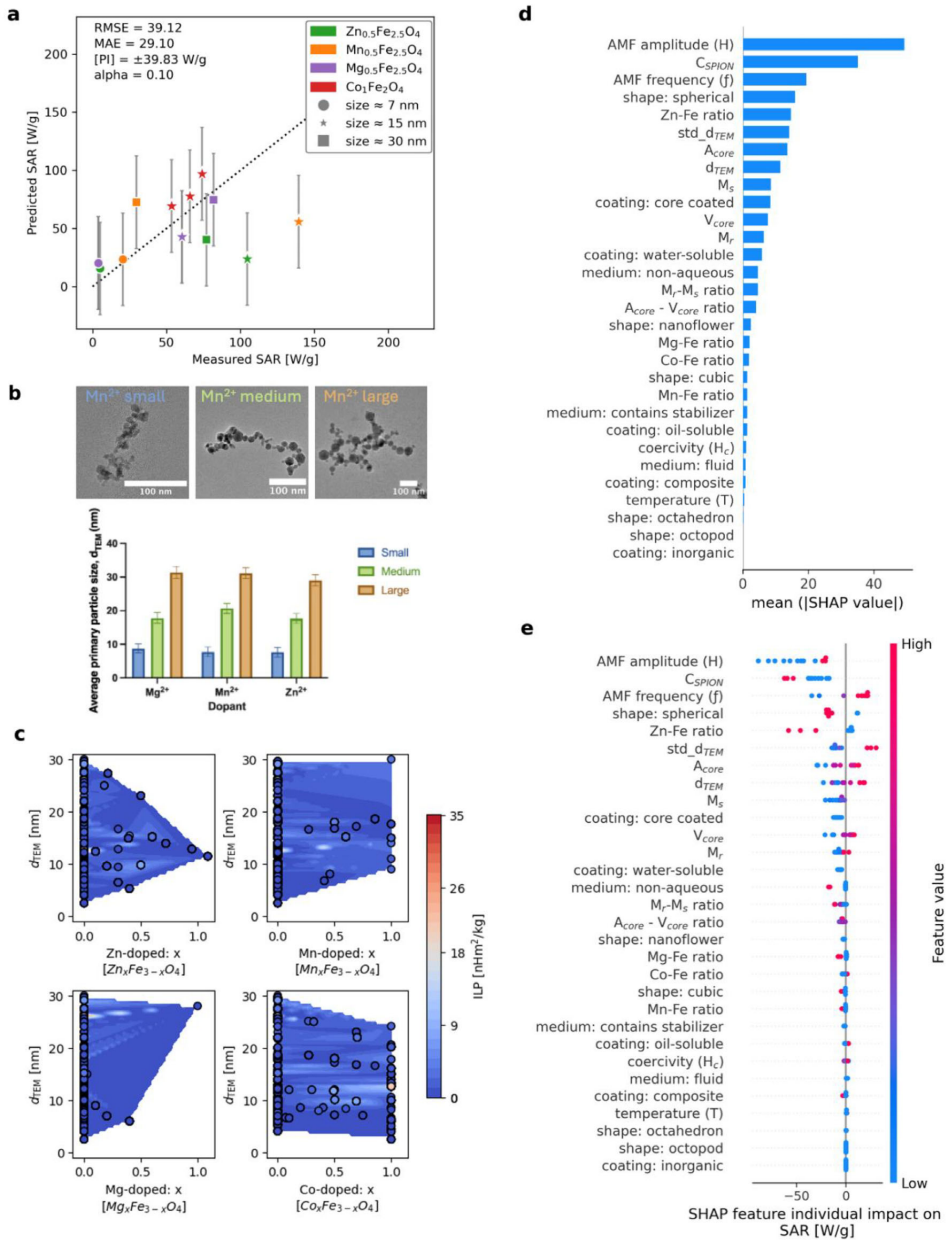
Overall CatBoost model performance and feature importance in predicting SAR for Zn‐, Mn‐, Mg‐, and Co‐doped SPIONs. (a) Comparison of predicted and measured SAR values. The conformal prediction intervals are detailed with grey error bars. (b) Transmission electron microscopy (TEM) micrographs presenting samples of Mg‐, Mn‐, and Zn‐doped nanoparticles with small (≈7 nm), medium (≈15 nm), and large (≈30 nm) particle sizes, respectively. The average primary particle size (*d*_TEM_) was calculated as the geometric mean of the diameters of 100 individual particles. (c) Contour plots detailing the relationship between particle size and dopant composition for nanoparticles in the training subset. Magnetic hyperthermia performance is represented as the intrinsic loss power (ILP), which is a normalized measurement obtained by dividing the SAR value by the square of the amplitude of the alternating magnetic field (*H* ^2^) times the frequency (ƒ). (d) Global SHAP analysis of the most important features affecting the predictive capabilities. (e) SHAP summary plot for the CatBoost model on the new unseen subset. The feature importance of the SAR prediction is displayed in the y‐axis from higher to lower importance. The locations of the red‐to‐blue‐colored dots on the x‐axis represent the positive or negative contribution of each feature on each instance in the model predictions. Red colored dots represent high feature values for a particular instance. Blue colored dots represent low feature values.

Based on this new dataset, Figure 8a shows the ability of the CatBoost model to predict SAR from this unseen data. In general, small‐size nanoparticles (≈7 nm) showed predictions that closely aligned with experimental SAR values. Particularly, Zn‐, Mn‐, and Mg‐doped nanoparticles showed the most accurate matches to the experimental SAR, showing predicted‐to‐measured differences as low as 3 W g^−1^. Moreover, among the small‐size nanoparticles, the largest observed discrepancy of Δ16 W g^−1^ was still nine times lower than the reported experimental discrepancies in the 0–250 W g^−1^ SAR prediction range [82].

Notably, the CatBoost model exhibited moderate prediction discrepancies for medium‐size nanoparticles (≈15 nm). Among these, Co‐ and Mg‐doped nanoparticles showed the most accurate agreement with the experimental SAR, with predicted‐to‐measured differences ranging from 12 to 23 W g^−1^. These values remain below the reported batch‐to‐batch variation of 39 and 143 W g^−1^ for spherical and cubic nanoparticles, respectively [21, 82]. In contrast, Zn‐ and Mn‐doped nanoparticles deviated significantly from the measured SAR, reporting differences as high as 81 and 83 W g^−1^, respectively. A similar trend was found for large‐sized nanoparticles (≈30 nm). The Mg‐doped nanoparticles reported the most accurate predictions, while Mn‐ and Zn‐doped nanoparticles reported noticeable deviations, reaching differences of Δ43 and Δ37 W g^−1^, respectively. These relatively large deviations for Zn‐ and Mn‐doped nanoparticles can be attributed to the limited availability of training data points at a doping fraction of *x* = 0.5 and *d*_TEM_ values of ≈15 nm and ≈30 nm (Figure 8c). Moreover, these large‐size nanoparticles are no longer in the superparamagnetic regime, which might contribute to higher predictive discrepancies in the model output. In contrast, Co‐doped nanoparticles showed lower prediction deviations at a doping fraction of *x* = 1 and a *d*_TEM_ value of ≈15 nm, likely due to the denser coverage of similar chemical and physical parameters in the training subset. Surprisingly, the Mg‐doped nanoparticles did not follow this pattern. Even with fewer data points, the medium‐ and large‐size nanoparticle predictions remained closer to the measured values.

It is important to emphasize that the performance of the CatBoost model is constrained by the distribution of particle size and dopant composition in the training set. The model provides more reliable predictions in regions where similar nanoparticles were represented, while predictions from unrepresented areas should be interpreted considering the lack of training data. Moreover, an additional limitation has to be considered due to the use of *d*_TEM_ to derive features such as *A*_core_ and *V*_core_. The approach applied to calculate morphological features might introduce systematic bias, particularly for complex shapes. This additional source of uncertainty should also be considered when the CatBoost model is applied to predict the SAR value of nanoparticles with complex morphologies, even when their size and dopant composition are inside the training domain. In addition, the categorical encoding of features such as particle shape, coating type, and suspension medium implies that the CatBoost model can only learn average effects associated with each category. These complex features are expected to modify the SAR value but remain poorly reported in the scientific literature. Therefore, the model predictions and feature‐importance analysis should be interpreted as reflecting dominant feature trends rather than as fully resolved feature descriptors.

The SHAP global barplot presented in Figure 8d confirms that extrinsic, physical, core shape, chemical, and magnetic properties maintain their relative importance rankings even when the CatBoost model is applied to newly collected data points. Furthermore, Figure 8e provides a SHAP beeswarm summary plot for the 12 doped nanoparticle samples, ranking predictive features by their influence on the model output. This plot also confirms that the trends observed among feature values and their corresponding SHAP values are maintained (Figure 7), indicating consistent feature impacts on SAR predictions across the new dataset.

Overall, these findings suggest that although the CatBoost model generalizes well to small‐sized nanoparticles (*≈*7 nm), further refinement is needed to improve predictions for medium and large‐sized SPIONs. To achieve this, expanding the training subset is essential for capturing a broader range of physicochemical properties. Several key descriptors were excluded in the current model due to poor reporting frequency in the literature, including magnetic anisotropy, crystallinity, magnetic diameter, hydrodynamic diameter, and zeta potential. An additional model limitation arises from the lack of systematically reported uncertainties for experimental features such as amplitude and frequency across the literature sources. Consequently, the CatBoost model prediction intervals reported in this study should be interpreted under the assumption of nominal input values.

This highlights both the need for more comprehensive characterization in experimental studies and the limitations of our model's accuracy when such features influence SAR. All data used to train the models in this study were derived from SAR measurements on colloidal SPION suspensions. Future work can explore correlations between colloidal and biological environments through the use of an in vivo dataset, potentially enabling predictive models for the behavior of magnetic nanoparticles under physiological conditions. Nevertheless, the developed CatBoost model can be employed to identify SPIONs with optimized properties, thereby reducing both experimental time and costs, and ultimately supporting the robust development of SPIONs for novel biomedical applications.

## 3 Conclusions

This study developed a predictive machine‐learning model for determining SAR based on the physical, chemical, and magnetic properties of SPIONs. A diverse dataset was compiled using natural language processing techniques and complemented by manual curation. Through Bayesian hyperparameter optimization, boosting‐based models, particularly CatBoost, demonstrated superior performance, achieving the lowest MAE and RMSE metrics alongside a high coefficient of determination (*R* ^2^ = 0.98). This indicates a robust capability to capture the complex dependencies influencing the hyperthermia performance of SPIONs. Further evaluation using conformal prediction analysis confirmed the reliability of the CatBoost model, which provided the narrowest prediction interval (±62 W g^−1^) among the evaluated models. Feature importance assessment via SHAP values highlighted the AMF amplitude and frequency as the most influential features, followed by SPION concentration and core surface area. Additionally, chemical properties, particularly the Zn‐Fe ratio, significantly impacted SAR predictions, with higher Zn─Fe ratios decreasing SAR and Co─Fe ratios increasing it. The model generalization capability was assessed using an independent dataset of SPIONs, accurately predicting SAR values for small‐sized nanoparticles (≈7 nm). However, predictions for medium (≈15 nm) and large‐sized (≈30 nm) SPIONs exhibited greater variability, suggesting the need for further model refinement in these size ranges. Overall, the CatBoost model serves as an important tool for predicting the hyperthermia performance of SPIONs, facilitating their design and optimization for biomedical applications such as cancer therapy.

## 4 Methods

### 4.1 Data Collection

The dataset was built in accordance with the preferred reporting items for systematic reviews and meta‐analyses (PRISMA) [48], using a four‐phase flow diagram composed of the following steps: identification, screening, eligibility, and inclusion.

The identification step was performed through an initial search of the SCOPUS database using a Boolean search string. The search string was designed to capture studies related to the application of superparamagnetic nanoparticles in hyperthermia treatments. The specific search terms used were: (“nanoparticle*” OR “iron oxide” OR “ferrite”) AND (“superparamagnet*” OR “magnet*”) AND (“hyperthermia” OR “heat*”). The search was limited to the publication years 1993–2023.

The screening step applied an NLP script developed in Python (Version 3.10) using the Natural Language Toolkit (NLTK) library (Version 3.8.1) and the Punkt pre‐trained model [100]. The script screened all the records obtained from the identification step and assigned a numeric value to each record based on the presence of relevant words in their titles and abstracts. The numeric value was determined through word‐to‐value mapping, where each relevant word was compared with the top 20 relevant words, word pairs, and word triplets extracted from an internal dataset, as detailed in Tables S7 and S8. The script then sorted the records according to their numeric values, selecting the top available 1000 scientific articles for further assessment.

The eligibility step used a second NLP script developed in the same Python environment. The script searched through all the screened full‐text articles to identify sentences containing a feature description or synonym, a numerical value, and a unit of measurement, as detailed in Table S9. The articles were then sorted by the number of occurrences of the target feature, with the script generating a heatmap to highlight which articles displayed the target and predictive features more frequently. All eligible scientific articles were manually curated to identify, confirm, or extract the numerical values of the 29 predictive features presented in this study. Most numerical values are detailed in figures, such as scatter plots, bar plots, and magnetization curves. WebPlotDigitizer software (Version 4.6) was employed to extract the numerical values from these figures [101].

### 4.2 Feature Engineering

The shapes of the SPIONs cores were classified into five mutually exclusive categories: cubic, spherical, nanoflower, octahedron, and octopod. Irregular and polyhedral shapes were classified as spherical when no other shape description was available in the article. The categorical features: “is cubic,” “is spherical,” “is nanoflower,” “is octahedron,” and “is octopod” were created using the “pd.get_dummies” function from the Pandas library (Version 2.0.3) [102]. This function converts categorical variables into a series of binary columns, where each column represents the presence of a specific shape with a value of one or the absence of that shape with a value of zero.

The average SPIONs core diameter (*d*_TEM_) was constrained to 30 nm or less to capture nanoparticles exhibiting superparamagnetic behavior. The surface core area (*A*_core_) and core volume (*V*_core_) were estimated based on the geometric shape of the SPION core. For the spherical cores, the SPIONs were modeled as symmetric spheres with a radius (*r*) equal to half that of the *d*_TEM_. The *A*_core_ and *V*_core_ were calculated using Equations 1 and 2, respectively.

(1) $$A_{\mathrm{core}}=4\pi {\left(\frac{d_{\mathrm{TEM}}}{2}\right)}^{2}$$

(2) $$V_{\mathrm{core}}=\frac{4}{3}\pi {\left(\frac{d_{\mathrm{TEM}}}{2}\right)}^{3}$$

For the cubic cores, the SPIONs were modeled as perfect cubes with an edge length (*l*) equal to that of the *d*_TEM_. The surface core area (*A*_core_) and core volume (*V*_core_) were calculated using Equations 3 and 4, respectively.

(3) $$A_{\mathrm{core}}=6{\left(d_{\mathrm{TEM}}\right)}^{2}$$

(4) $$V_{\mathrm{core}}={\left(d_{\mathrm{TEM}}\right)}^{3}$$

For the nanoflower cores, the SPIONs were modeled as clusters composed of 12 identical non‐overlapping spheres surrounding one central sphere, generating a cluster composed of 13 identical spheres with radius (*r*) [103, 104] The radius (*r*) of each individual sphere was defined as one‐sixth that of the *d*_TEM_. The surface area (*A*_core_) of the cluster was calculated as the sum of the surface areas of the 12 outer spheres using Equation 5. The total cluster volume (*V*_core_) was calculated as the sum of the volumes of all 13 spheres using Equation 6.

(5) $$A_{\mathrm{core}}=12*4\pi {\left(\frac{d_{\mathrm{TEM}}}{6}\right)}^{2}$$

(6) $$V_{\mathrm{core}}=13*\frac{4\pi }{3}{\left(\frac{d_{\mathrm{TEM}}}{6}\right)}^{3}$$

For the octahedron cores, the SPIONs were modeled as regular octahedrons with eight faces shaped as equilateral triangles. It was assumed that these octahedrons lay on one of their faces (*F*_ADE_), with the adjacent face (*F*_DEF_) forming an external angle *β*_1_ with the surface, as illustrated in Figure S10a. The edge length (*l*) of the octahedron was calculated from the *d*_TEM_ measurements using Equation 7. It was assumed that *d*_TEM_ was equal to the sum of the length of the symmetry line AC and the projected line CF*’*. The surface area (*A*_core_) and volume (*V*_core_) of the octahedral cores were subsequently calculated as detailed in Equations 8 and 9, respectively.

(7) $$l=\frac{d_{\mathrm{TEM}}}{\sqrt{3/4}\;\times \;\left(1+\cos\left(\beta_{1}\right)\right)}$$

(8) $$A_{\mathrm{core}\;}=2\sqrt{3}l^{2}$$

(9) $$V_{\mathrm{core}\;}=\frac{\sqrt{2}}{3}l^{3}$$

For the octopod cores, the SPIONs were modeled as the spatial intersection of a central cube with eight equilateral tetrahedrons positioned at the cube vertices. Three vertices of each tetrahedron were oriented to intersect the three nearest midpoints of the edges of the cube, resulting in a partial overlap with the cube, as detailed in Figure S10b. The edge length (*l*) of each tetrahedron was calculated from *d*_TEM_ measurements using Equation 10. It was assumed that the *d*_TEM_ was equal to the sum of *l* and the length of the projection lines *T'S* and *A'R*. Where *β*_2_ is the internal angle between the cube's top surface and the tetrahedron face *F*_ABC_. The core surface area (*A*_core_) was determined by summing the exposed surface areas of tetrahedrons and the cube, as shown in Equation 11. The core volume (*V*_core_) was calculated as the total volume of the central cube and the eight tetrahedrons by subtracting the overlapping volume, as detailed in Equation 12.

(10) $$l=\frac{d_{TEM}}{\left(1+2\;\times \;\sqrt{3/4}\;\times \;\cos\left(\beta_{2}\right)\;\right)}$$

(11) $$A_{\mathrm{core}\;}=6l^{2}+8\frac{3}{4}\sqrt{3}l^{2}\;$$

(12) $$V_{\mathrm{core}}={\left(\frac{2l}{\sqrt{2}}\right)}^{3}+8\frac{l^{3}}{6\sqrt{2}}-\frac{2l^{3}}{3\sqrt{2}}$$

The SPION coating characteristics were standardized into five non‐mutually exclusive features: surface coating, oil‐soluble coating, water‐soluble coating, and inorganic coating. Binary values were assigned to each feature based on their physicochemical properties, chemical nature, the presence or absence of a coating, and whether the coating material was composed of one or multiple chemical substances.

The chemical composition of the SPIONs was derived from the general formula of doped magnetite reported in each scientific article. This formula was represented as M_x_Fe_3−x_O_4_, where “M” denotes the dopant element, classified into four types: Zn, Co, Mn, and Mg. The fraction of iron cations replaced by a dopant is defined as “x.” Typically, the value of “x” was obtained from the reported theoretical SPIONs general formula. However, when a publication provided a more precise measurement technique, such as inductively coupled plasma (ICP) analysis, the empirically determined “x” value was considered. Maghemite SPIONs (γ‐Fe_2_O_3_) could not be represented within the same compositional framework, as it is a distinct iron oxide phase and is typically undoped. Including maghemite would require a separate representation strategy, which could introduce inconsistencies in feature definition and reduce model generalizability. Furthermore, magnetite‐based nanoparticles (M_x_Fe_3−x_O_4_) account for 90% of the reported ferrites used for biomedical applications, while only 10% use maghemite [28]. Given both its limited representation in literature and the need for consistent feature definition within a uniform compositional framework, maghemite nanoparticles were excluded from the current study [105].

Magnetic features, including saturation magnetization (*M*_s_) and remanence (*M*_r_), were extracted from scientific articles in various units, such as emu g^−1^, emu g_Fe_^−1^, emu cm^−3^, Am^2^ kg^−1^, and kA m^−1^. All units were standardized to emu g^−1^ to assess consistency and comparability. To convert emu cm^−3^ and kA m^−1^ to emu g^−1^, an average SPION density of 5 g cm^−3^ was employed [25]. The equivalence of 1 Am^2^ kg^−1^ corresponds to 1 emu g^−1^ to was also utilized [105]. To convert emu g_Fe_^−1^ to emu g^−1^, the iron content of the SPION was calculated using the formula M_x_Fe_3−x_O_4_. Where “M” denotes other possible metal substitutions, and “Fe_3−x_” represents the varying iron content. Remanence values reported as negligible were considered as 0 emu g^−1^. The coercivity (*H*_c_) was also extracted in different units of measurement, including Oersted (Oe), militesla (mT), and kA m^−1^. To ensure consistency, all measurements were standardized to Oe. Equivalences of 1 mT correspond to 0.7958 kA m^−1^, and 1 mT corresponds to 10 Oe were also applied [105]. The temperature at which the magnetic properties were measured was recorded in Kelvin (K). A value of 300 K was assumed if the text “room temperature” was specified, and no other numerical value was provided in the scientific article.

SAR values reported in the literature were expressed along different units such as W g^−1^ nanoparticle, W g^−1^ Fe, W g^−1^ total metal. These values were systematically normalized to W g^−1^ nanoparticle by using the general formula M_x_Fe_3−x_O_4_ [g mol^−1^]. Specifically, the Fe mass was derived from the Fe_3−x_ fraction. The metal mass corresponded to the contribution of Fe_3−x_ and M_x_. The following atomic weights were used to perform the unit conversion: Zn = 65.4 g mol^−1^, Co = 58.9 g mol^−1^, Mn = 54.9 g mol^−1^, Mg = 24.3 g mol^−1^, and Fe = 55.84 g mol^−1^.

### 4.3 Data Splitting Strategy, Normalization, and Exploratory Analysis

The data stratification process employed a combination of shape, doping, and coating features to create a composite stratification label. The “apply” function in Pandas [66] was utilized to derive the “shape_label,” “doped_label,” and “coating_label” columns based on predefined conditions for each feature category. These labels were then concatenated into a unified “stratify_label,” representing unique combinations of shape, doping, and coating attributes as detailed in Table S3. The class weights were calculated by a five‐step algorithm. In the first step, the algorithm counts how many samples exist per class and uses the reciprocal value of these counts to allocate an initial weight. In the second step, it normalizes the weights by dividing each weight by the minimum value among them. In the third step, the algorithm applies a logarithmic transformation (log1p) to all the normalized weights to reduce extreme variations among them. In the fourth step, a second normalization is applied by dividing again each log‐transformed weight against the minimum weight value among them. Finally, the algorithm allocates each normalized log‐transformed weight to their respective instance according to their stratified label.

Subsequently, the “train_test_split” function from the Scikit‐Learn library [71] (Version 1.3.2) was applied in a three‐stage process, stratified by the “stratify_label.” This procedure generated the training (70%), validation (10%), calibration (10%), and test (10%) subsets, ensuring proportional representation across all stratification categories. A fixed random seed (42) was applied to maintain reproducibility throughout the splitting process. Each subset underwent preprocessing with the “MinMaxScaler” class from the Scikit‐Learn library, which performed feature scaling within the range of −1 to 1. The scaler was fitted to the training subset to prevent data leakage.

The distribution of all features was visualized using the “violinplot” and “subplot” functions from the Matplotlib library [106] (Version 3.8.4). The comparative analysis between predictive features was conducted using the “PCA” and “TSNE” functions from the Scikit‐Learn library, with the “n_components” parameter set to two for both functions. Furthermore, the “random_state” parameter of the “TSNE” function was set to 42 to ensure reproducibility.

### 4.4 Model Bayesian Hyperparameter Optimization

The ML models LR, RR, ENR, PLS, SVR, KNN, DTR, and RFR were developed using the Scikit‐Learn library [61] (Version 1.3.2). The boosting‐based models were implemented by applying the CatBoost library [107, 108] (Version 1.2.5), the LightGBM library [109] (Version 4.1.0), and the XGBoost library [110] (Version 2.0.3). The DNN model was developed by applying the TensorFlow library [111] (Version 2.15.0).

The optimal hyperparameter values for each model were established by applying the “create_study” function and the “optimize” method from the Optuna library [68] (Version 3.5.0). Table S4 details the range of hyperparameter values sampled through an optimization process composed of 1000 iterations. The wRMSE values from validation and training subsets were calculated and plotted along all the iterations. The gap between wRMSE values was calculated as the difference among wRMSE values from the validation and the training subsets.

The criteria for selecting the optimal hyperparameters for each model were established by a four‐step algorithm. In the first step, the algorithm identifies the lowest wRMSE value from the validation subset and sets a threshold that is 10% higher than this value. In the second step, it filters the hyperparameter configurations from the 1000 iterations to retain only those whose validation wRMSE values fall below this threshold. In the third step, the algorithm sorts the remaining configurations by prioritizing those with smaller gap values. Finally, in the fourth step, it selects the hyperparameter configuration that exhibits the lowest overall gap value calculated from the validation and training subsets.

The optimal number of epochs for training the DNN model was determined by analyzing the performance changes along the learning curves, as detailed in Table S10 and Figure S8c. The wRMSE on the validation subset was selected as the loss function. The search for the optimal epoch count was constrained to the 400–590 range, where the wRMSE on the validation subset had reached a plateau. The lowest gap value inside this range was applied as a criterion to select the optimal number of epochs.

A stop callback mechanism was employed during the optimization of the CatBoost, LightGBM, and XGBoost models using the “early_stopping” method and “early_stopping_rounds” parameter, respectively. This callback terminated the training process if the reduction in the wRMSE values on the validation data did not improve after 30 iterations in the LightGBM model, and 20 iterations in both the CatBoost and the XGBoost models. The plot that details the overall wRMSE reduction for the 12 ML models was elaborated by applying the “subplot” and “scatter” functions from the Matplotlib library [106] (Version 3.8.4).

The “importance.get_param_importances” function from the Optuna library was utilized to identify the top five most important hyperparameters in each ML model. The hyperparameter optimization scatterplots of each machine learning model were generated using the “get_cmap,” “add_gridspec,” “add_subplot,” and “scatter” functions from the Matplotlib library. The y‐axis limits were standardized across all subplots ranging from 0 to 1 measured in transformed SAR units [W g^−1^, Yeo‐Johnson].

### 4.5 Model Performance Comparison

The performance of the optimized models was assessed through an analysis of *R* ^2^ and a comparative box plot analysis of the MAE. This evaluation process included generating SAR predictions from the test subset and comparing them with the experimental SAR values obtained from the scientific literature. Furthermore, the evaluation involved calculating several regression metrics on both the validation and training subsets, including *R* ^2^, MAE, MedAE, RMSE, and wRMSE. These metrics were computed using Equations (13), (14), (15), (16), and 17, respectively.

(13) $$R^{2}=1-\frac{\sum_{i=1}^{n}{\left(y_{i}-{\hat{y}}_{i}\right)}^{2}}{\sum_{i=1}^{n}{\left(y_{i}-\bar{y}\right)}^{2}}$$

(14) $$\mathrm{MAE}=\frac{1}{n}\sum_{i=1}^{n}\left|y_{i}-{\hat{y}}_{i}\right|$$

(15) $$\mathrm{MedAE}\;=\;\mathrm{median}\;\left(\left|y_{i}-{\hat{y}}_{i}\right|\right)$$

(16) $$\mathrm{RMSE}=\sqrt{\frac{1}{n}\sum_{i=1}^{n}{\left(y_{i}-{\hat{y}}_{i}\right)}^{2}}$$

(17) $$\mathrm{wRMSE}\;=\sqrt{\frac{\sum_{i=1}^{n}w_{i}{\left(y_{i}-{\hat{y}}_{i}\right)}^{2}}{\sum_{i=1}^{n}w_{i}}}$$

where y_i_ is the experimental SAR value [W g^−1^]; ${\hat{y}}_{i}$ is the predicted SAR value [W g^−1^]; $\bar{y}$ is the SAR mean value from experimental measurements [W g^−1^]; *n* is the total number of data points; “median” represents the middle value of the absolute error sorted list. *w*_i_​ represents the weight assigned to each data point based on its class or importance.

 The MAE, and *R* ^2^ metrics were obtained by applying the “mean_absolute_error” and “r2_score” functions from the Scikit‐Learn library (Version 1.3.2). A modified version of the “mean_squared_error” function from the Scikit‐Learn library was also applied to calculate the RMSE metric. The “median” function from the Numpy library (Version 1.26.4) was used to calculate the MedAE metric [109, 111].

### 4.6 Model Agglomerative Hierarchical Analysis

The heatmap displaying the Spearman correlation coefficient was elaborated by applying the functions “clustermap” and “corr” from the libraries Seaborn (Version 0.13.2) and Pandas (Version 2.2.2), respectively. The permutation importance scores were calculated using the “premutation_importance” function from the Scikit‐Learn library (Version 1.3.2). The comparative plots were elaborated by applying the “subplots” and “barh” functions from the Matplotlib library (Version 3.8.4), as well as the “scatterplot” function from the Seaborn library [112]. The *R* ^2^ metric was calculated using the “r2_score” function from the Scikit‐Learn library.

### 4.7 Model Conformal Prediction Analysis and Feature Importance Analysis

The prediction intervals (PI) were calculated by applying the class “MapieRegressor” from the library Mapie (Version 0.8.3) [113]. The SHAP values were obtained through the class “shap.Explainer” from the library SHAP (Version 0.47.2). The SHAP global bar plots and beeswarm summary plots were created by applying the function “shap.summary_plot” with the parameter “plot_type” set to “bar” or “dot,” respectively, in separate function calls.

### 4.8 Transmission Electron Microscopy (TEM)

The reported Zn‐, Mn‐, and Mg‐doped ferrites were synthesized by flame spray pyrolysis following the protocol described in our previous study [39]. The doped ferrites were collected from the same synthesis batch, and additional morphological characterization was performed using TEM. The doped ferrites were prepared for TEM characterization by dispersing them through sonication in ethanol at a concentration of 0.01 mg mL^−1^. Sonication was carried out for 5 min at a 90% amplitude using a cup horn ultrasonicator (Sonics, USA), supplemented with a 10 s vortex mixing every 1 min. A 5 µL droplet of each sample was placed on a Formvar/Carbon 300 square mesh copper grid (Delta Microscopies, France). Transmission electron microscopy (TEM) was used to visualize the particles using a FEI Titan Themis 200 equipment (Thermo Fisher Scientific, USA) operating at 200 kV. ImageJ software was used to measure the particle size of 100 particles. Histograms were plotted using the Sturges method [114]. The Shapiro‐Wilk test for normality was used to determine that the particle size distributions of all samples were log‐normally distributed. The geometric mean and geometric standard deviation from the fitting of the log‐normal curve were used to determine the major particle sizes.

## Author Contributions

E.R.V‐C. collected, analyzed, and interpreted the data and drafted the initial manuscript. S.R.A. analyzed and interpreted the data and was the major contributor in writing the manuscript. J.Z. analyzed and interpreted the data and contributed to reviewing the manuscript. Y.d.C.S.‐L. performed the TEM analysis and contributed to reviewing the manuscript. A.T. supervised the study design, interpreted the findings, and substantially revised the manuscript. P.L. supervised the study design and extensively revised the manuscript. All authors read and approved the final manuscript.

## Conflicts of Interest

The authors declare no conflicts of interest.

## Code Availability

The code that supports the findings of this study is available at https://doi.org/10.17044/scilifelab.29835419.

## Supporting information




**Supporting File**: smll72569‐sup‐0001‐SuppMat.docx.

## Data Availability

The dataset supporting this study's findings is available at https://doi.org/10.17044/scilifelab.29835419.
