# Supplementary material for: Machine Learning on Systematically Curated Data Reveals Key Determinants of Magnetic Hyperthermia Performance
Source: Small. 2026 Jan 30;22(17):e10453. doi: 10.1002/smll.202510453 (PMC13003278; doi:10.1002/smll.202510453)

**Supplementary Information:**

Machine learning on systematically curated data reveals key determinants of magnetic hyperthermia performance

Edgar Régulo Vega-Carrasco^1^, Shaquib Rahman Ansari^1^, Jiaxi Zhao^2^, Yael del Carmen Suárez-López^1^, Per Larsson^2,*^ and Alexandra Teleki^1,*^

^1^Department of Pharmacy, Science for Life Laboratory, Uppsala University, 75123 Uppsala, Sweden

^2^Department of Pharmacy, Uppsala University, 75123 Uppsala, Sweden

[*alexandra.teleki@scilifelab.uu.se](mailto:*alexandra.teleki@scilifelab.uu.se) and per.r.larsson@uu.se


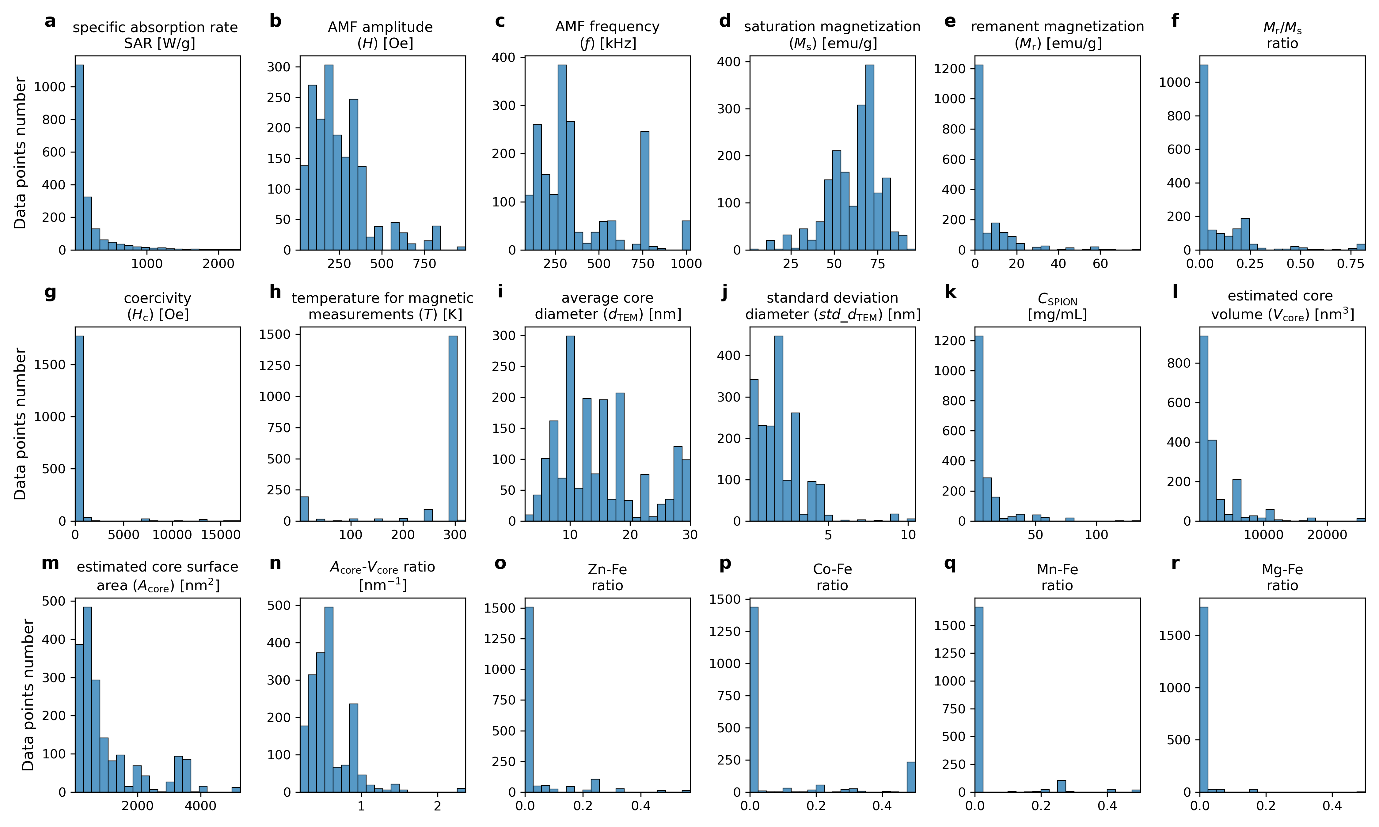

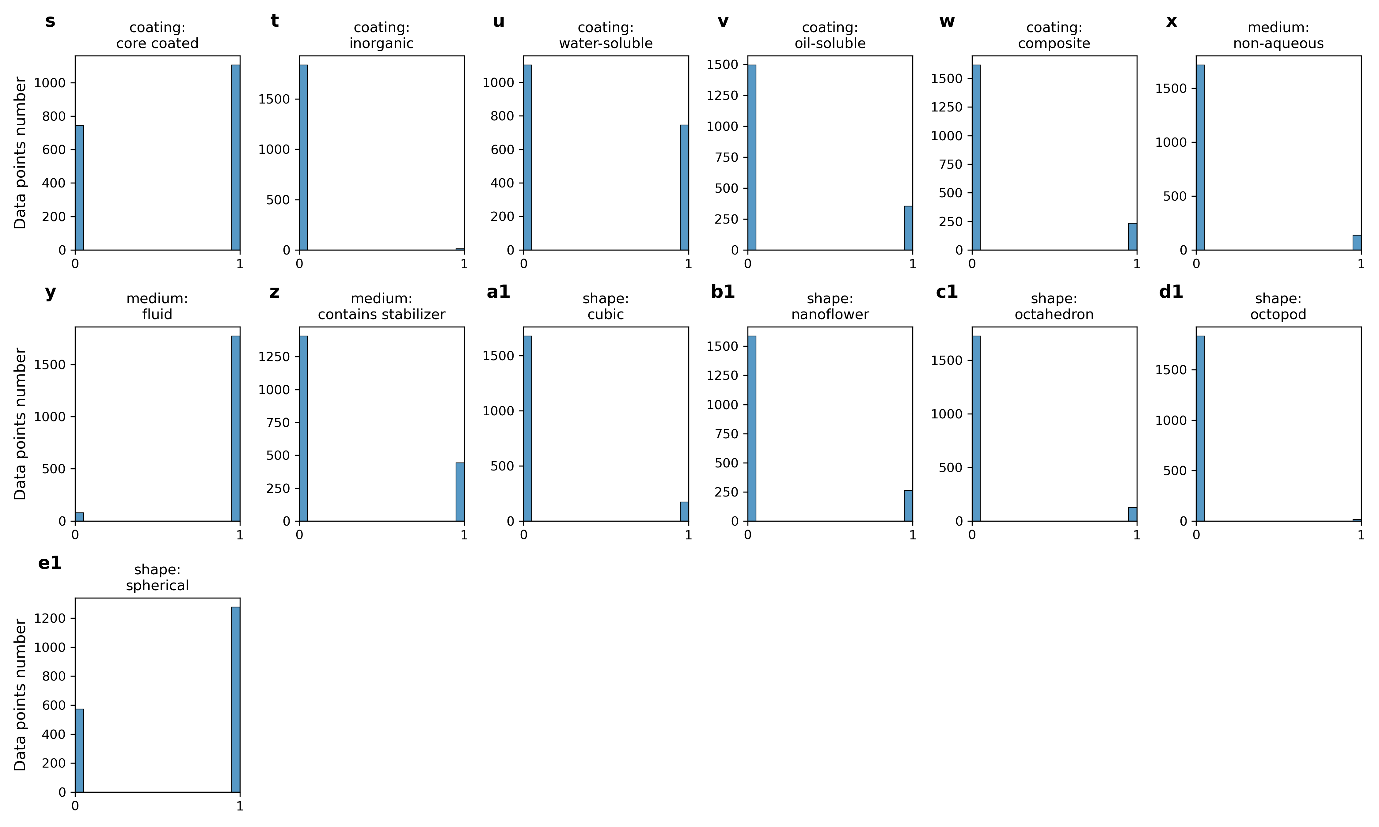


**Figure S1**. Dataset feature distribution. **Numerical features**: Panel (a) presents the distribution of the target feature. Panels (b) through (r) display the distribution of the predictive numerical features. **Categorical features:** Panels (s) through (e1) show the distribution of the predictive categorical features. For all subplots, the y-axis represents the numerical values of the data points within each bin. The x-axis represents the value of numerical features expressed in their respective unit of measurement. The x-axis of numerical features indicates the presence (1) or absence (0) of the feature.


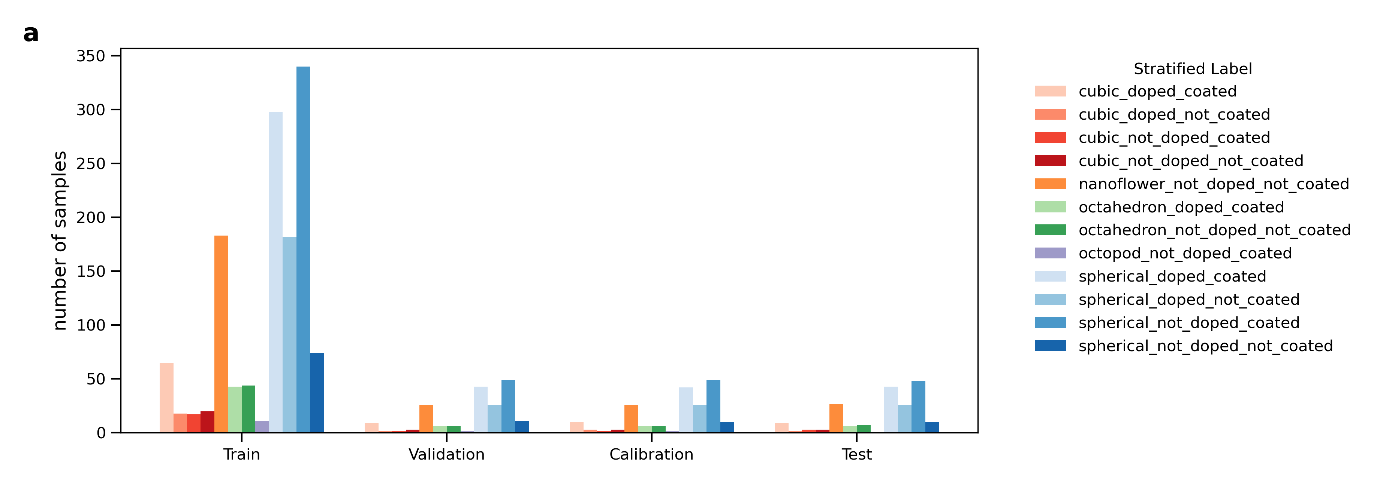


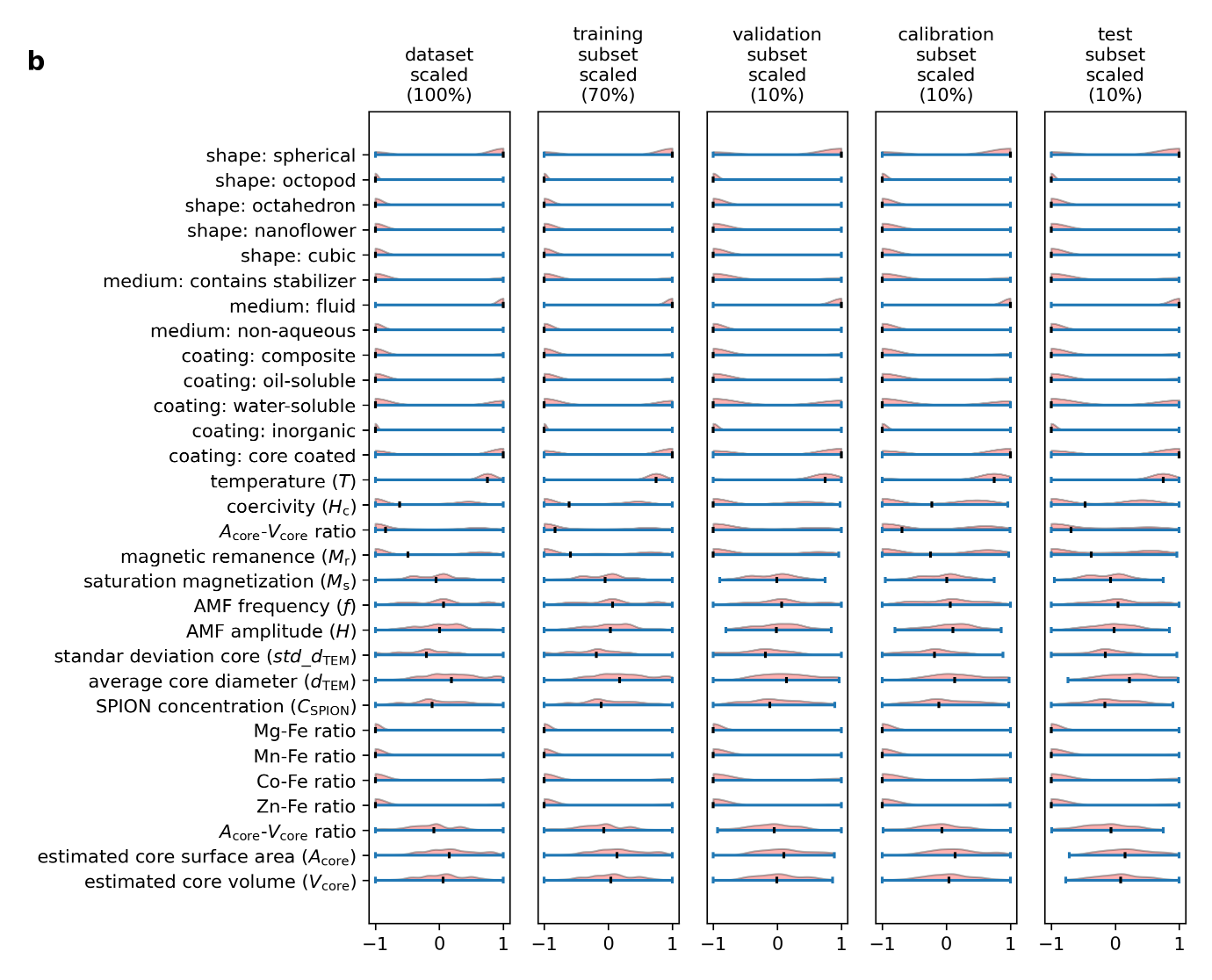


**Figure S2**. (a) Distribution of stratified labels across the four subsets. (b) Distribution of dataset predictive features after transformation and normalization. All numeric features were transformed by applying the Yeo-Johnson method. Then, the min-max scaling technique was applied to both numerical and categorical features to distribute their values from -1 to 1. The y-axis displays the predictive features. The data distribution of each feature is represented as a half-violin density plot colored in light red. The black vertical line inside each density plot represents the median value of each feature.


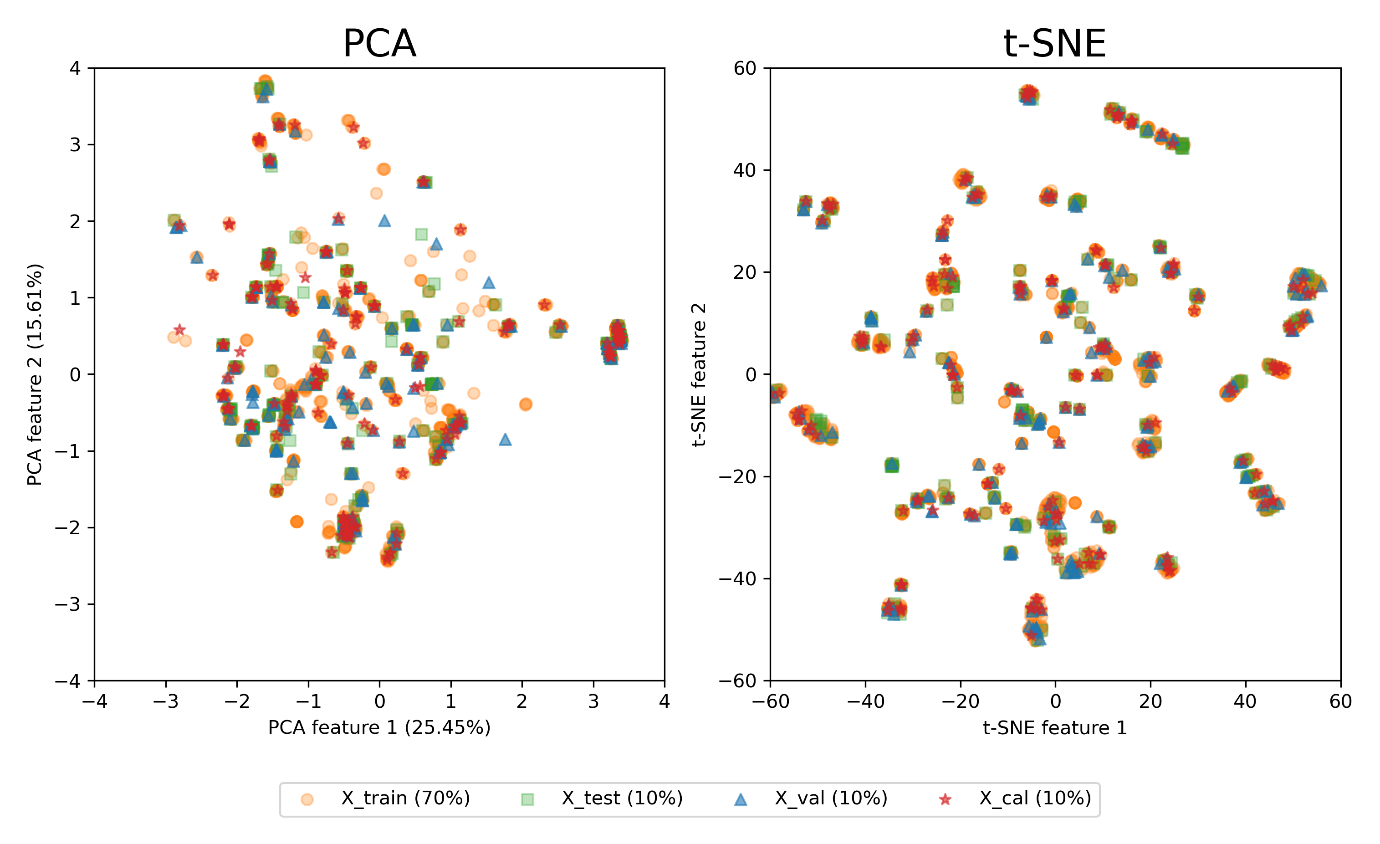


**Figure S3.** Principal component analysis (PCA) against t-distributed stochastic neighbor embedding (t-SNE). Training, test, validation, and calibration data instances are represented by yellow circles, green squares, blue triangles, and red stars, respectively.


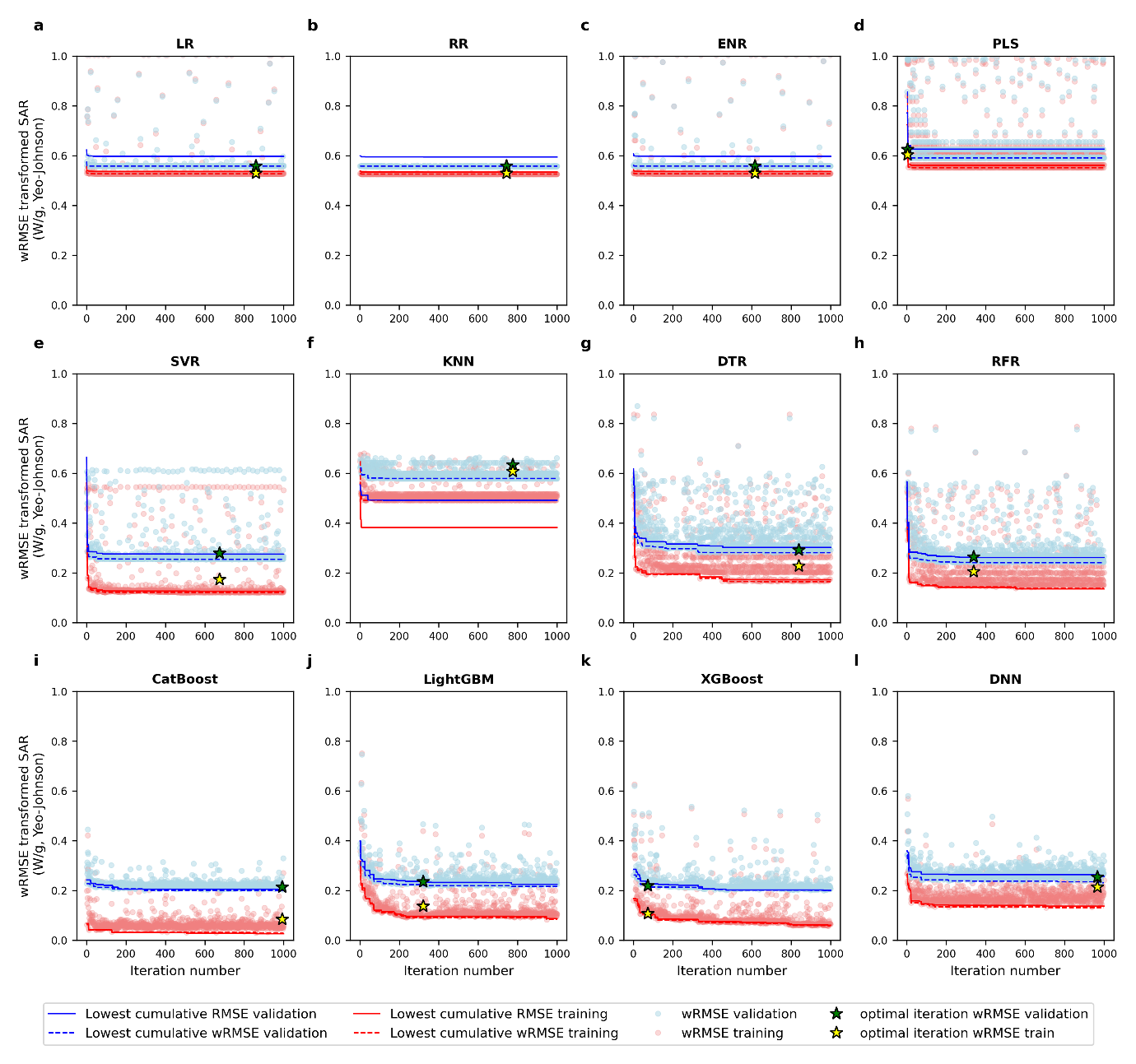


**Figure S4.** Bayesian hyperparameter optimization of twelve machine-learning models. The y-axis presents the weighted root mean squared error (wRMSE) values, measured in transformed SAR units [W/g, Yeo-Johnson], which are valid for all subplots. The x-axis, located at the bottom of each panel, indicates the iteration number of the optimization process, and it is valid for all subpanels. wRMSE measurements from the validation and training subsets are detailed as light blue and light red dots, respectively. The lowest cumulative wRMSE results from the validation and training subsets are represented by blue and red dotted lines, respectively. Similarly, the lowest cumulative RMSE results from the validation and training subsets are represented by blue and red lines, respectively. Green and yellow stars detail the wRMSE values obtained from the validation and training subsets that fulfil the established criteria for optimal hyperparameter selection. Panels (a) through (d) illustrate the optimization outcomes for linear regression-based models. Panels (e) through (l) detail the optimization results for non-linear regression-based models.


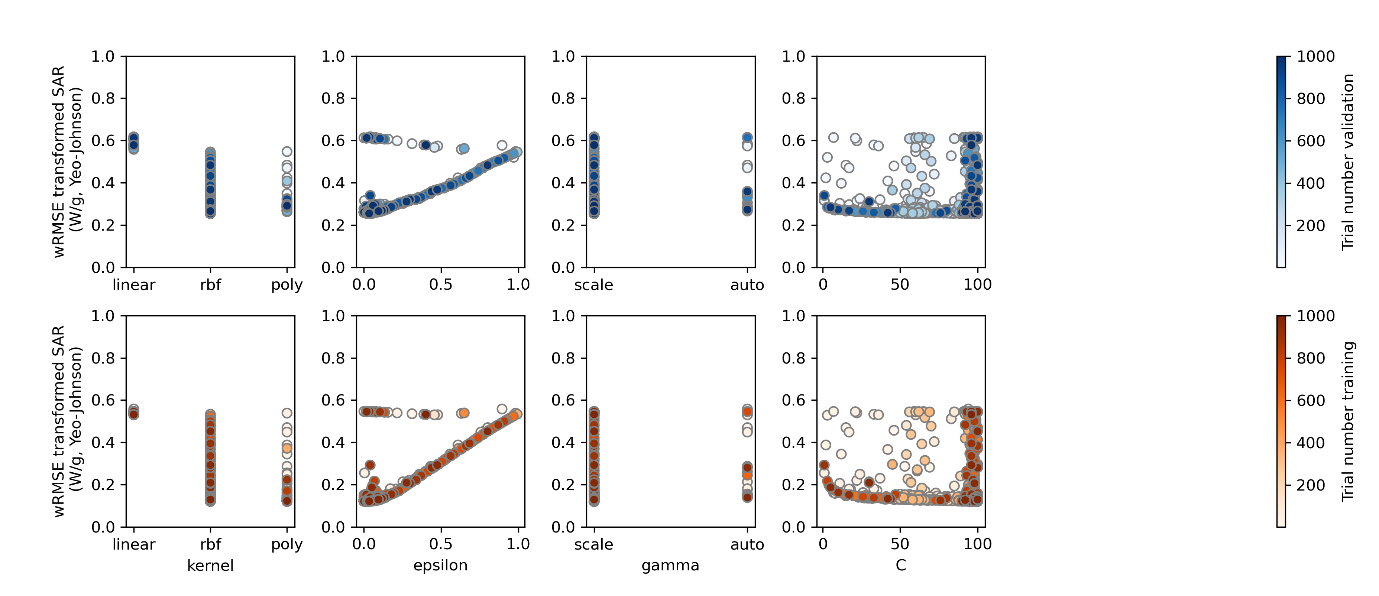


**Figure S5.** Hyperparameter Bayesian optimization of the support vector regressor model. Validation and training measurements are represented by light blue and light orange dots, respectively. As the trial number increases, the intensity of the light orange and light blue-colored dots increases. The weighted root mean squared error (wRMSE) value, measured in transformed SAR units [W/g, Yeo-Johnson], is displayed on the y-axis, and it is valid for all the subplots. The x-axis at the bottom of each panel displays the respective model hyperparameters.


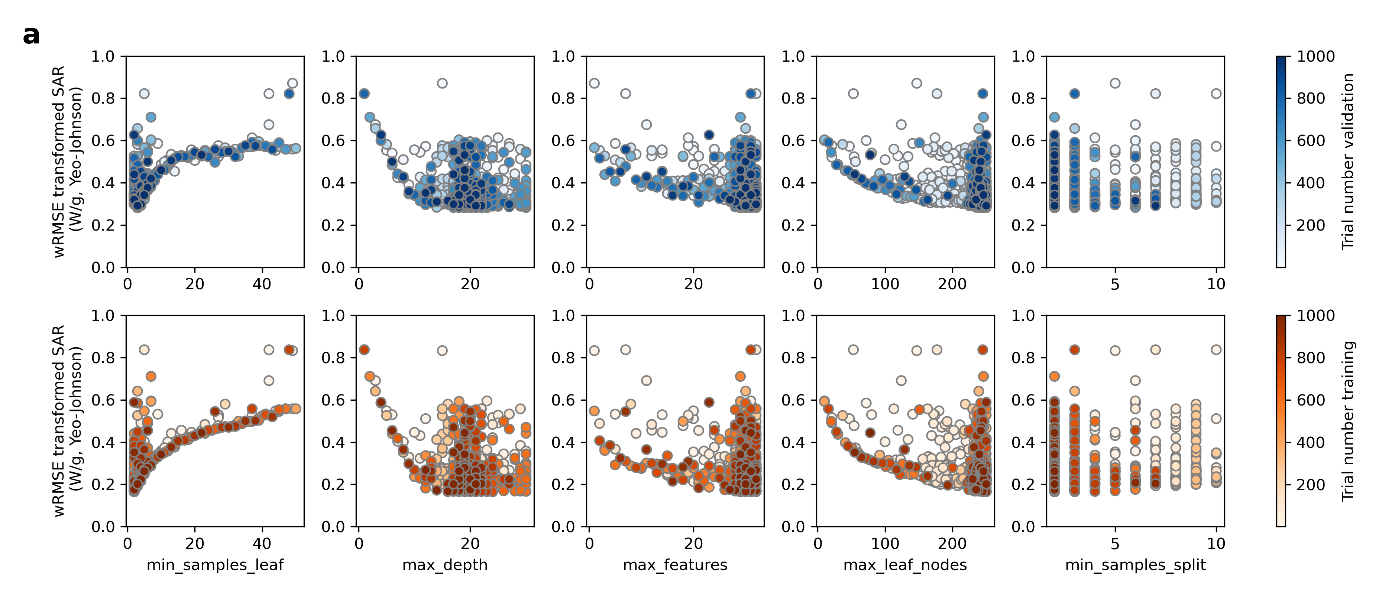

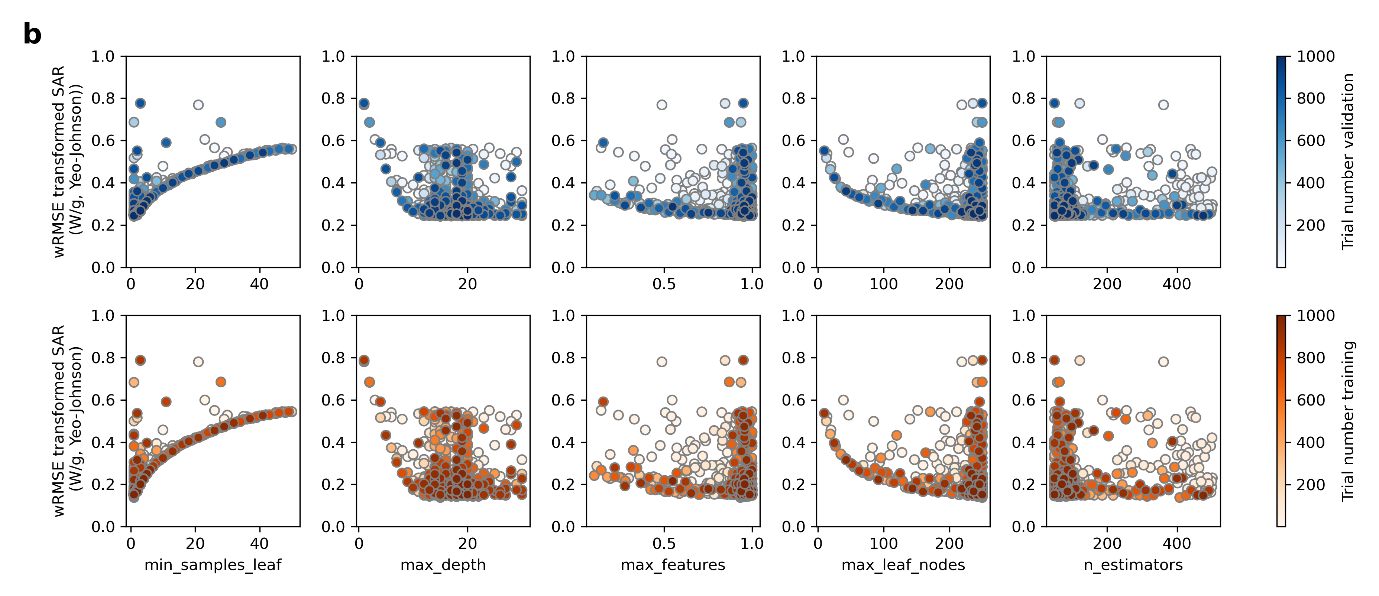


**Figure S6.** Hyperparameter Bayesian optimization of tree-based models: Decision tree regressor model (a) and random forest regressor model (b). Validation and training measurements are represented by light blue and light orange dots, respectively. As the trial number increases, the intensity of the light orange and light blue-colored dots increases. The weighted root mean squared error (wRMSE) value, measured in transformed SAR units [W/g, Yeo-Johnson], is displayed on the y-axis, and it is valid for all the subplots. The x-axis on the bottom of each panel displays the respective model hyperparameters.


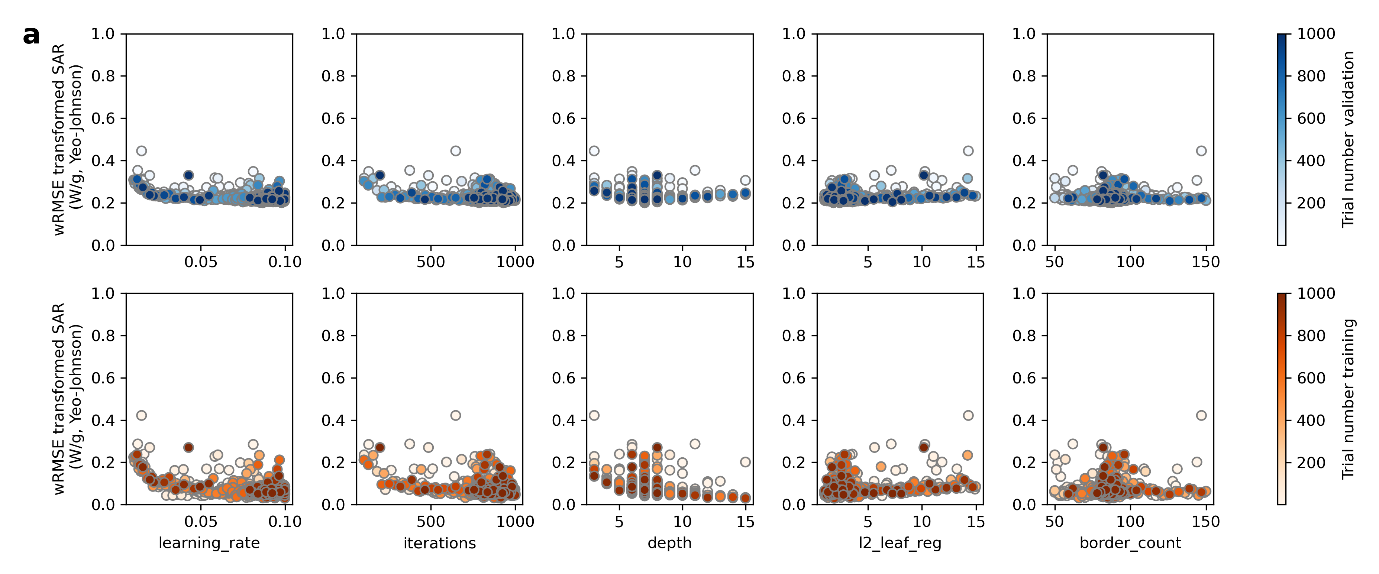

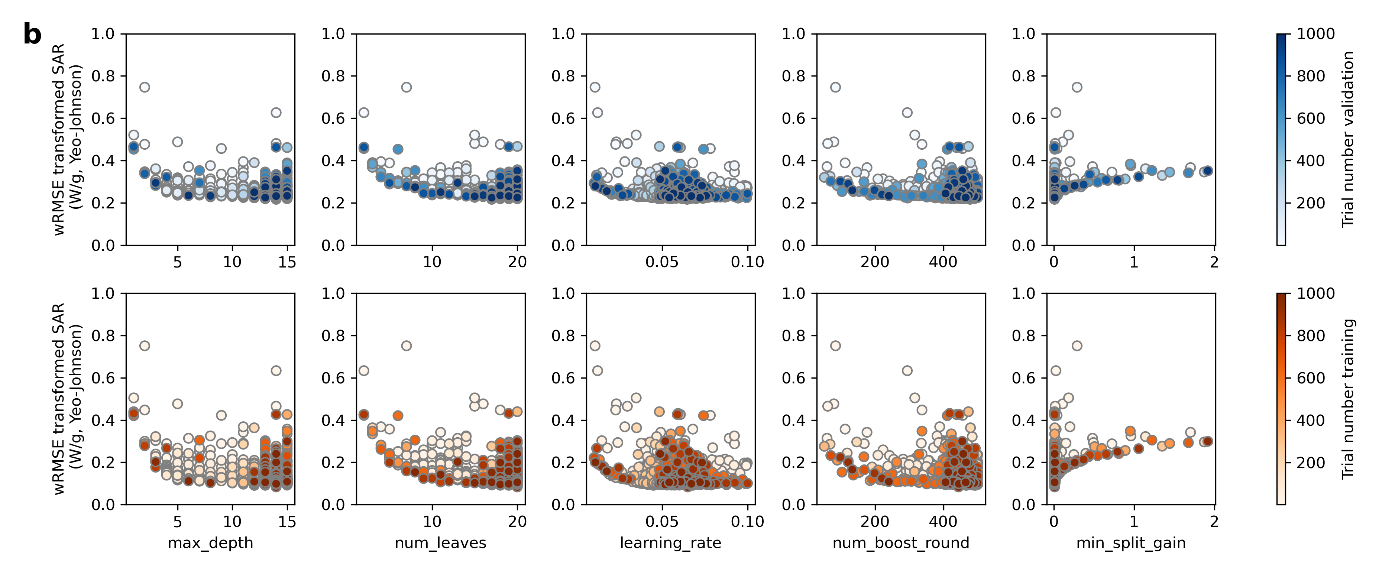

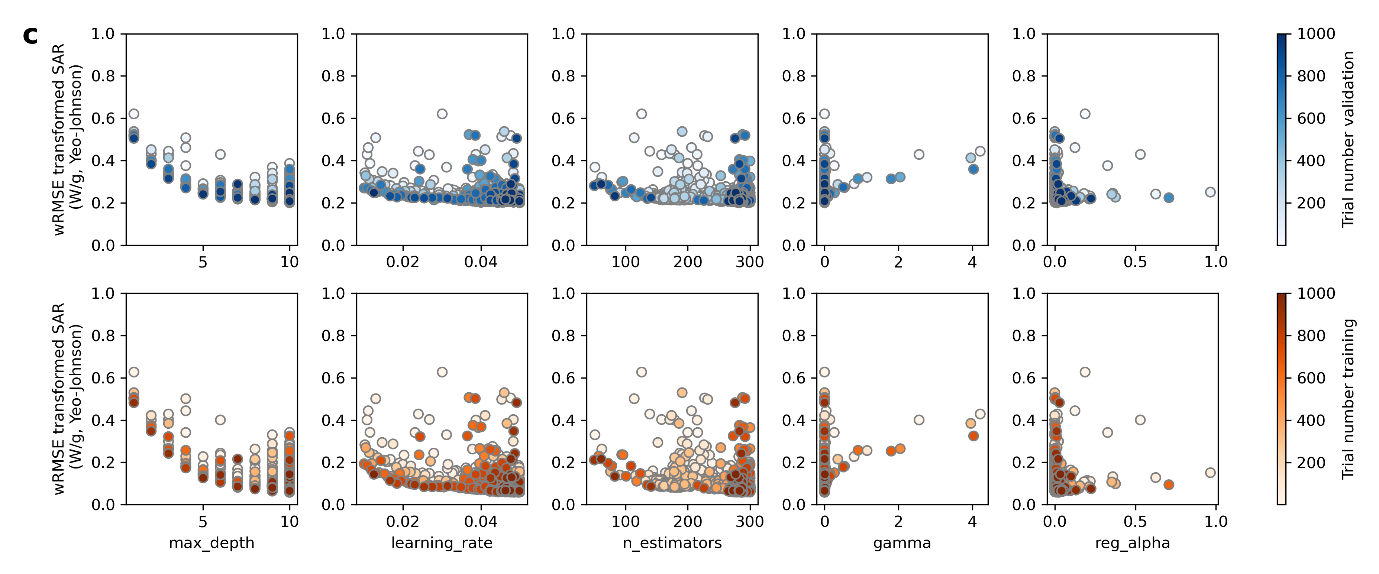
**Figure S7.** Hyperparameter Bayesian optimization of boosting-based models: (a) categorical boosting regressor model, (b) light gradient boosting model, and (c) extreme gradient boosting model. Validation and training measurements are represented by light blue and light orange dots, respectively. As the trial number increases, the intensity of the light orange and light blue-colored dots increases. The weighted root mean squared error (wRMSE) value, measured in transformed SAR units [W/g, Yeo-Johnson], is displayed on the y-axis, and it is valid for all the subplots. The x-axis on the bottom of each panel displays the respective model hyperparameters.


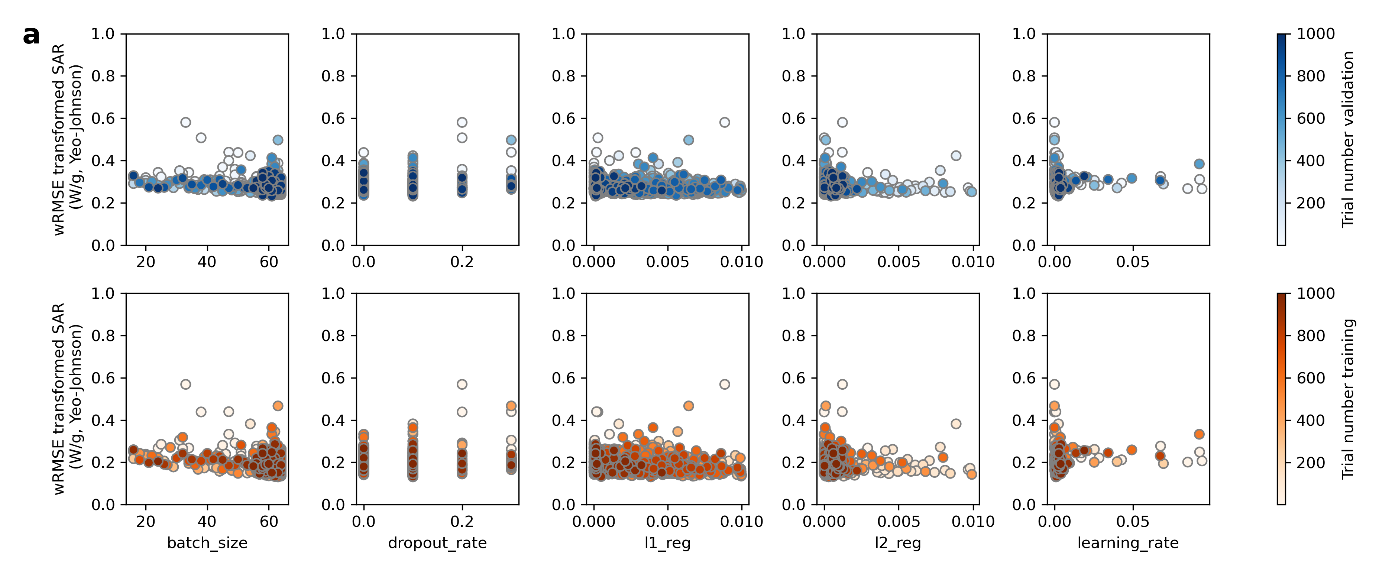

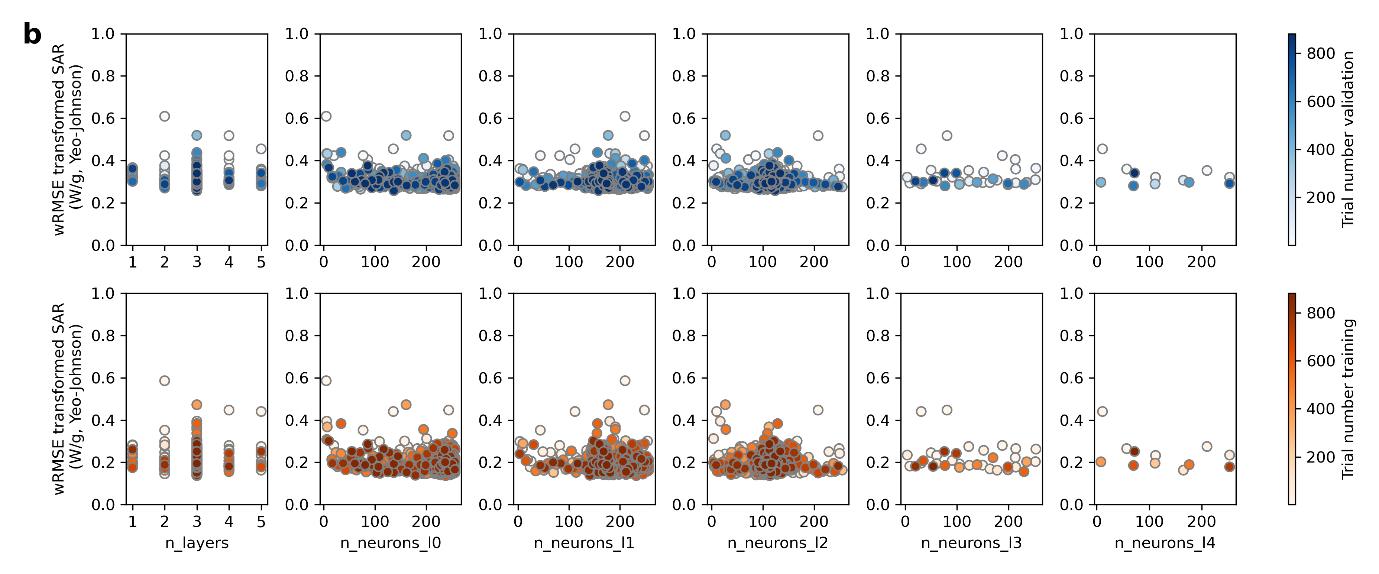

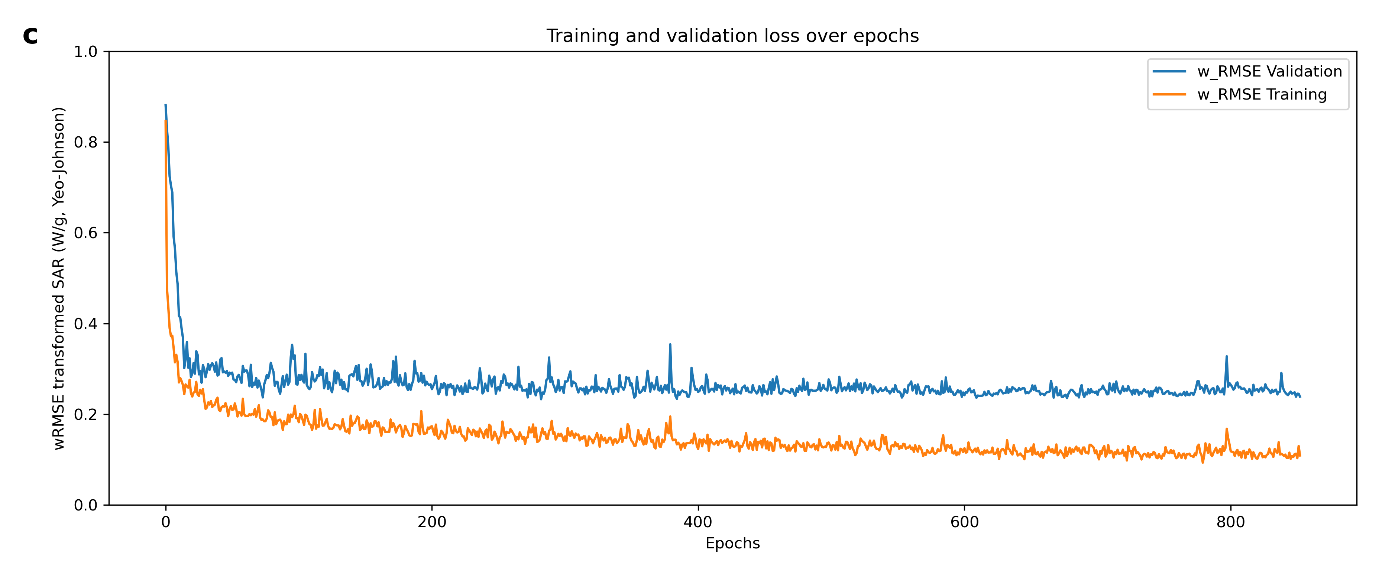
**Figure S8**. (a) Hyperparameter Bayesian Optimization of the deep neural network model. (b) Neural network architecture design. Validation and training measurements are represented by light blue and light orange dots, respectively, in panels a and b. As the trial number increases, the intensity of the light orange and light blue-colored dots increases. (c) Learning curve to determine the optimal number of epochs. The weighted mean squared error value, measured in transformed SAR units [W/g, Yeo-Johnson], is displayed on the y-axis, and it is valid for all the subplots. The x-axis displays the respective model hyperparameter values in panel a, the number of layers and nodes per layer in panel b, and the number of epochs in panel c.


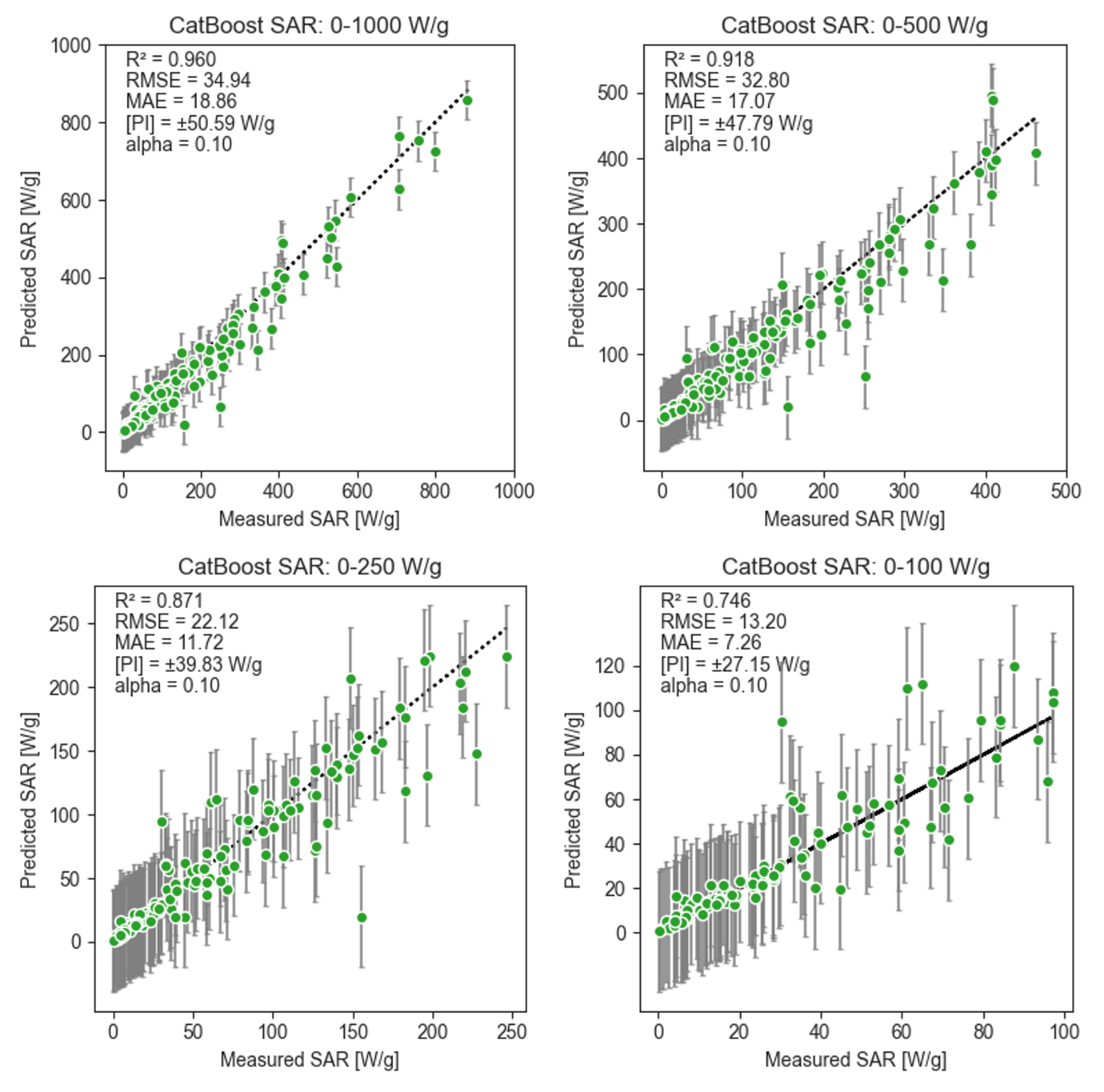


**Figure S9.** Prediction Intervals (PI) calculated from the CatBoost model across four expected SAR output ranges: 0-100, 0-250, 0-500, 0-1000 (SAR, W/g).


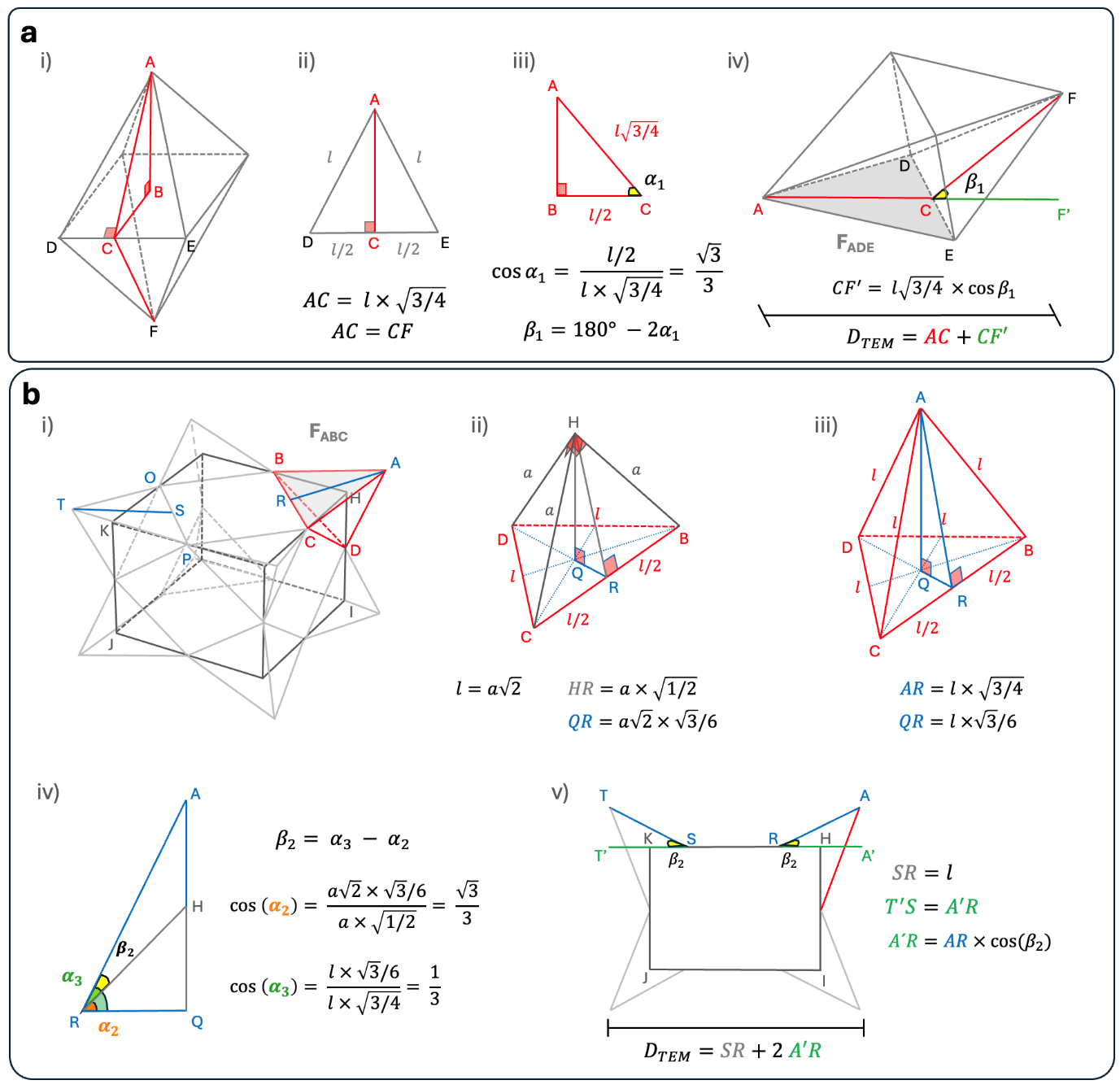


**Figure S10.** Modelling of octahedron and octopod SPION cores. Panel (a) comprises four subpanels illustrating the geometrical and mathematical modelling of octahedron cores. Subpanel (i) shows a regular octahedron with edge length (*l*). Subpanel (ii) displays the calculation of the lengths of symmetry lines AC and CF. Subpanel (iii) explains the method used to determine the values of the angles $\alpha_{1}$ and $\beta_{1}$. Subpanel (iv) details how the octahedron face F_ADE_ and the adjacent face F_DEF_ form the angle $\beta_{1}$ with the surface and illustrates the calculation of the length of the projected line CF’. The length of the average core diameter (D_TEM_) was assumed to be equal to the sum of the AC and CF’ lines. Panel (b) is composed of five subpanels that detail the geometrical and mathematical modelling of octopods cores. Panel (i) shows a symmetrical octopod composed of a central cube and eight equilateral tetrahedron with edge length (*l*). Subpanels (ii) and (iii) detail the calculation of the lengths of symmetry lines HR, QR, and AR. Subpanel (iv) explains the method applied to obtain the values of the angles $\alpha_{2}$,$\alpha_{3}$, and $\beta_{2}$. Subpanel (v) explains the calculation of the projected lines T’S and A’R and the line SR. The length of the average core diameter (D_TEM_) was assumed to be equal to the sum of the SR and 2 times the A’R line.

**Table S1.** Selection of predictive features based on the variables defined by the linear response theory and determination of SAR*.

| **Physical Phenomena** | **Equation** | **Parameters** | **Number** |
| --- | --- | --- | --- |
| The Néel (*τ*_N_) relaxation | $\tau_{N}=\tau_{0}e^{\frac{KV_{M}}{kT}}$ | τ_0_=10^−9^ s  *K*: anisotropy constant  *V_M_*: magnetic core volume  *k*: the Boltzmann constant  *T*: absolute temperature  *η*: viscosity of the medium  *V_H_*: hydrodynamic volume | (1) |
| The Brownian (*τ*_B_) relaxation | $\tau_{B}=\frac{3{\eta V}_{H}}{kT}$ |  | (2) |
| The overall relaxation time (τ) | $\tau=\frac{\tau_{B}\tau_{N}}{\tau_{B}{+\tau}_{N}}$ |  | (3) |
| Rosensweig’s power dissipation equation (*P*) | $P=\pi\mu_{0}\chi_{0}H^{2}f \frac{2\pi f\tau}{1+{(2\pi f\tau)}^{2}}$ | *μ*_0_: the permeability of free space  χ_0_: is the magnetic susceptibility  *H*: field strength of AMF  *f* : magnetic field frequency | (4) |
| Experimental  Specific Absorption Rate  (SAR) | $SAR=C_{p}\times\frac{m_{s}}{m_{n}}\times\frac{dT}{dt}$ | dT/dt: initial slope of the heating curve  *m*_s_: mass of the suspension  *m*_n_: mass of the nanoparticles  *C*_p_: specific heat capacity of the suspension | (5) |

*: Development of SPIONs for magnetic hyperthermia is often guided by the linear response theory (LRT). This theoretical model not only explains the mechanisms of heat dissipation but also provides the basis for how particle and field parameters influence heating efficiency. According to LRT, heat dissipation from magnetic nanoparticles in an AMF is caused by the delay in the relaxation of their magnetic moment, through either the rotation within the particle (Néel) or the rotation of the particle itself (Brownian). The Néel (*τ*_N_) and Brownian (*τ*_B_) relaxation times are given by Eq. 1 and 2. Typically, SPIONs used for magnetic hyperthermia have broad size distribution, resulting in simultaneous occurrence of both Brownian and Néel processes. The overall relaxation time τ is then described by Eq. 3. The heat dissipation value is calculated using the harmonic average of both relaxations and their relative contributions and expressed by Rosensweig’s power dissipation equation (Eq. 4). Experimentally, the SAR of SPIONs is calculated from the heating profiles of the magnetic fluid according to Eq. 5. Based on the equations (Eq. 1-5) the heat released from SPIONs in an AMF is directly linked to intrinsic properties such as particle volume, anisotropy constant, hydrodynamic volume, susceptibility, and extrinsic properties such as viscosity of medium, and AMF parameters. These variables directly informed the selection of features used in our ML model

**Table S2.** Categorization criteria applied to the different types of SPIONs coating materials.

**
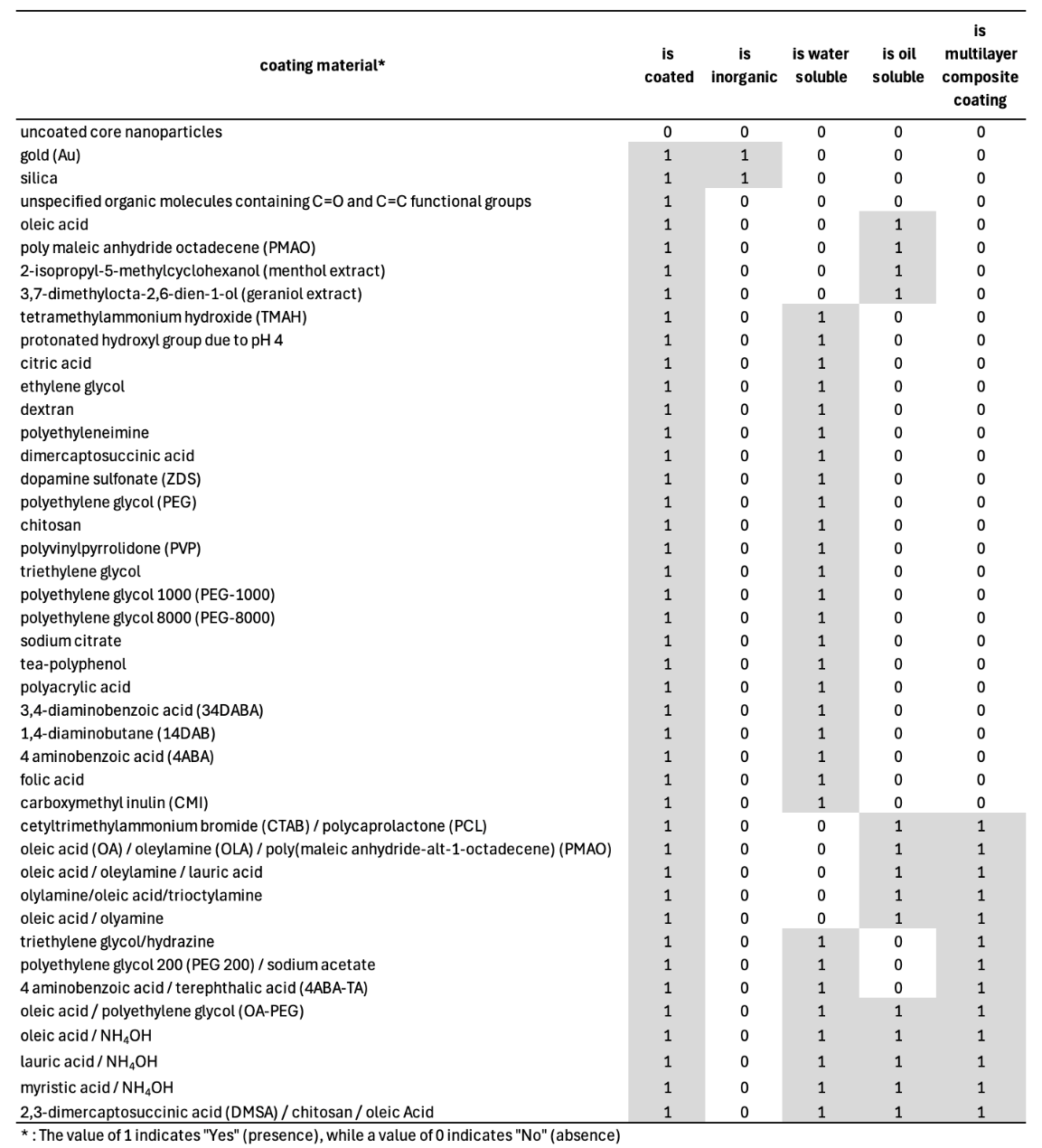
**

**Table S3.** Categorization criteria applied to the different types of suspension medium where hyperthermia measurements were performed.

**
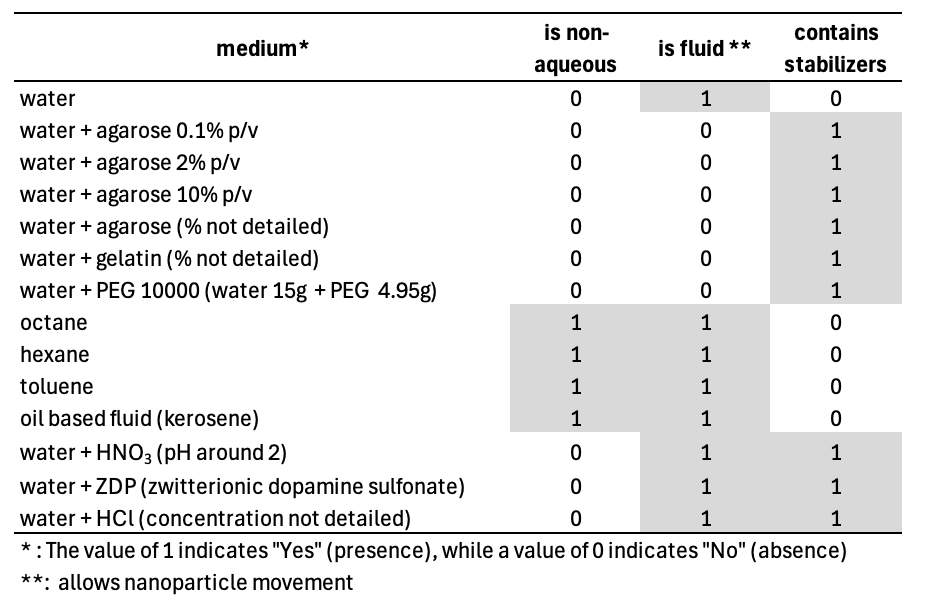
**

**Table S4.** Distribution of stratified labels, class counts, and class weights from the training subset.

**
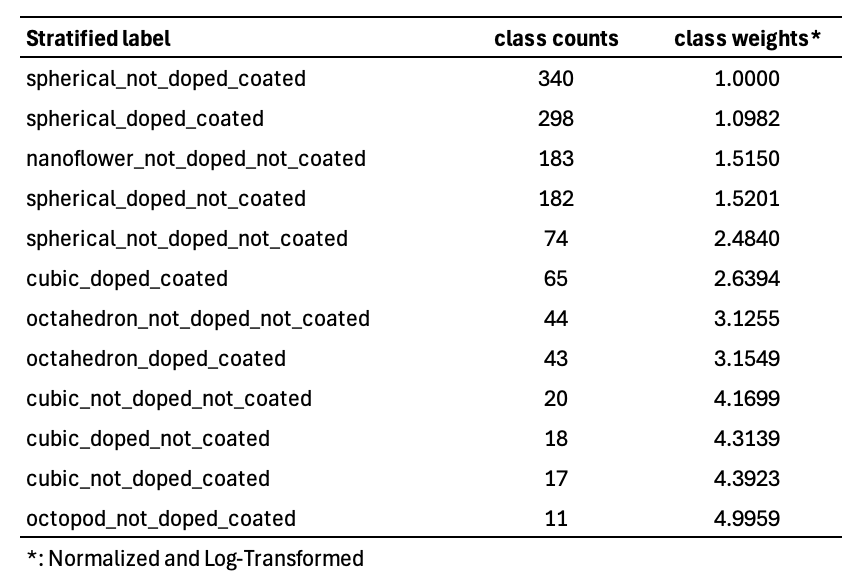
**

**Table S5.** Hyperparameter search space for Bayesian optimization. Optimal parameters within each model are detailed in the optimal output column.


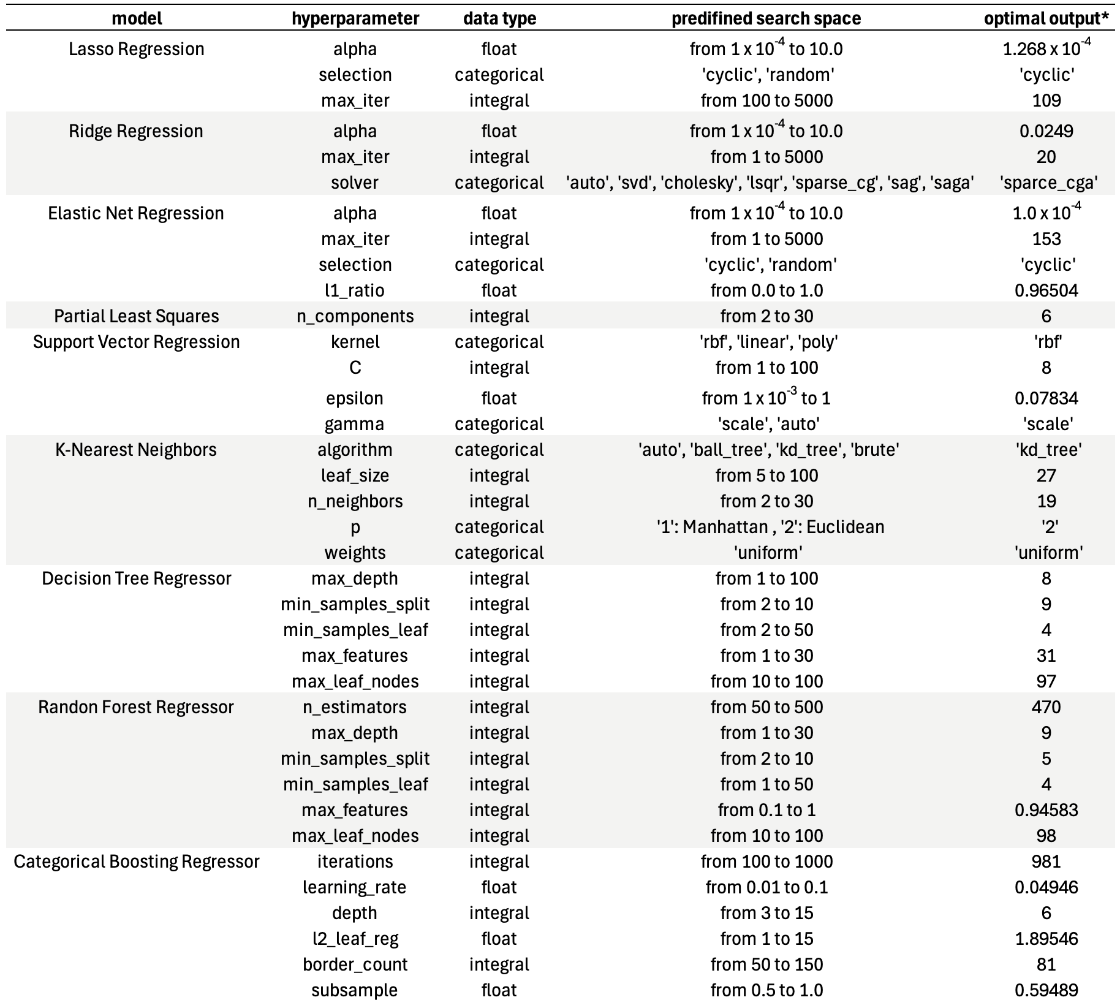

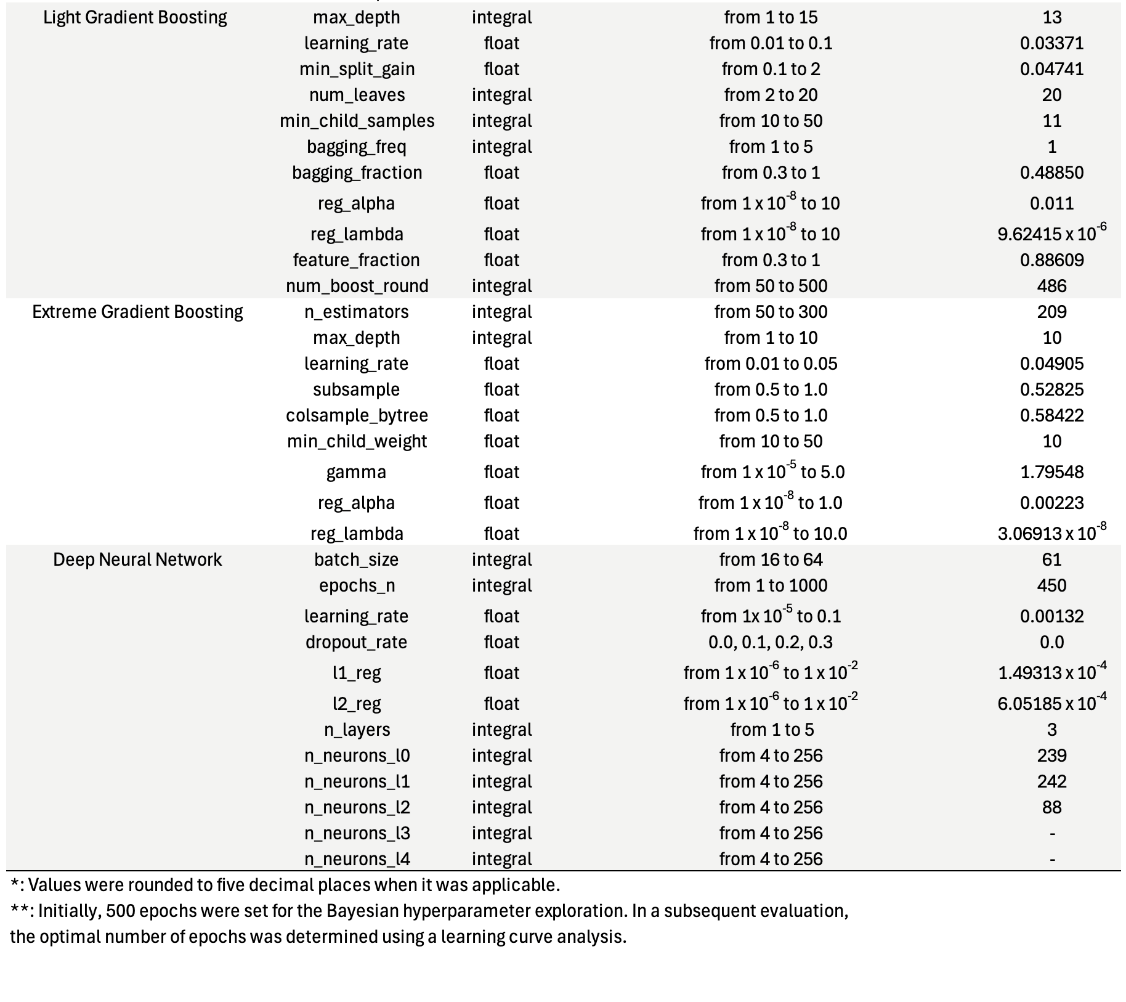


**Table S6.** Model predictive performance after hyperparameter tuning.


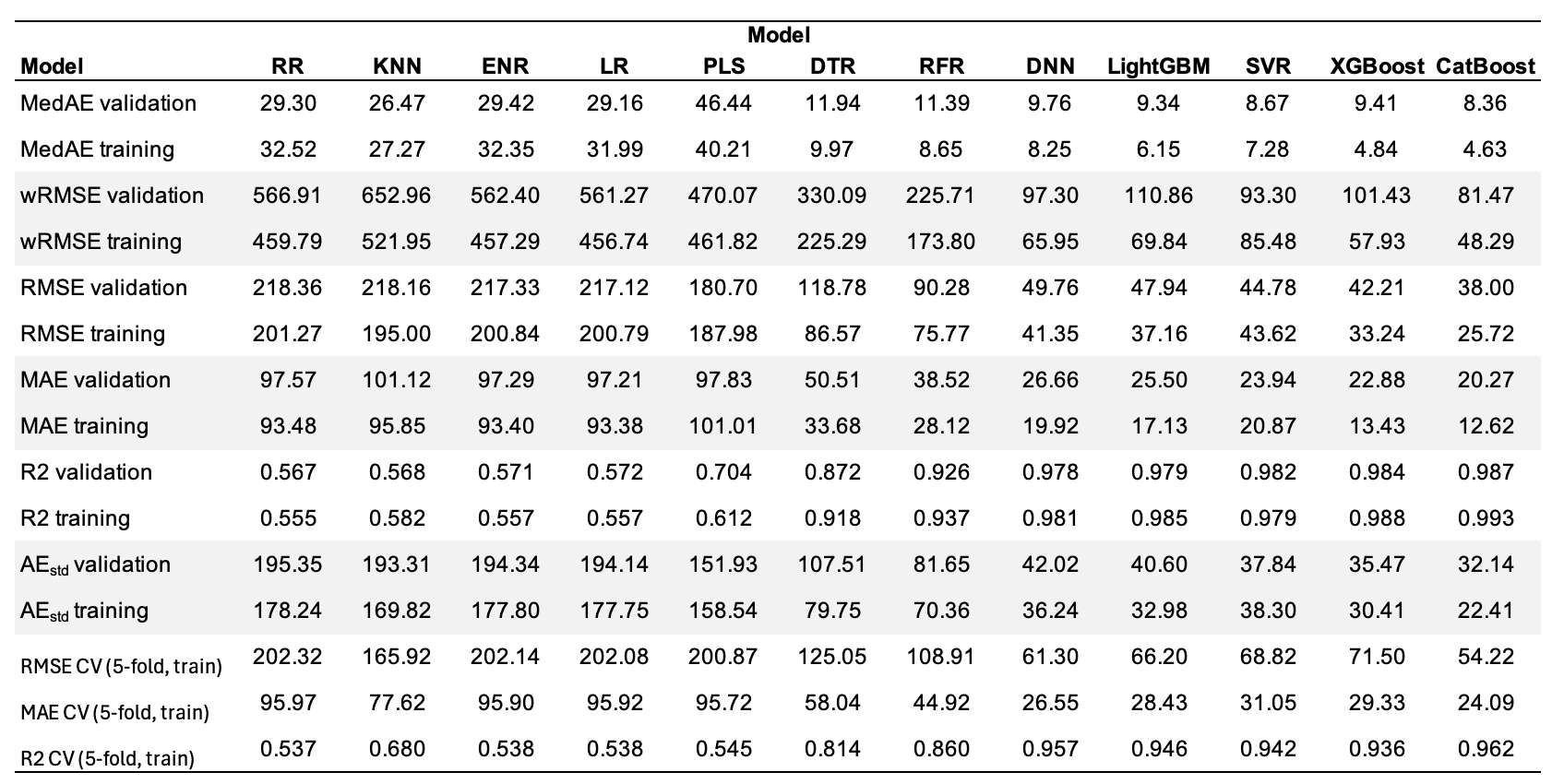


**Table S7.** Physical, chemical, and magnetic properties of nanoparticles from the new unseen dataset. In addition, both measured and predicted hyperthermia performance values are provided.


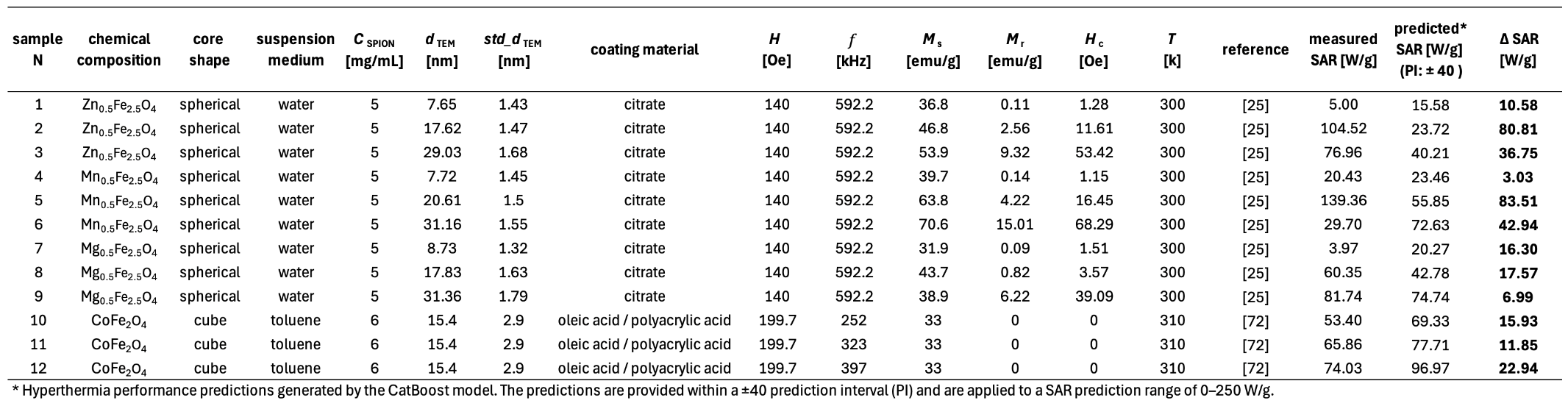


**Table S8.** Top 20 Word, word pair, and word triplet extracted from the titles and abstracts of the internal database of relevant scientific articles.


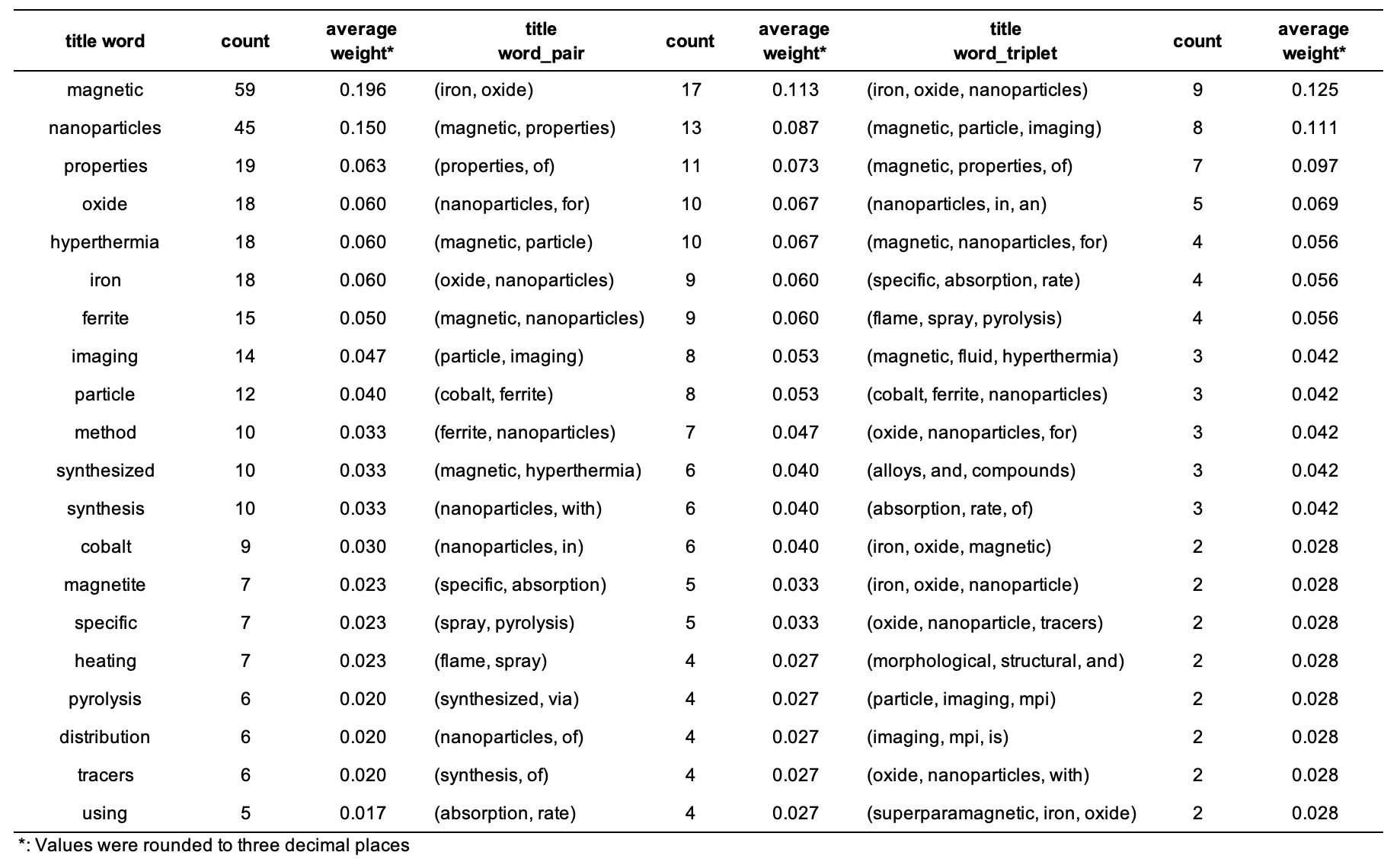

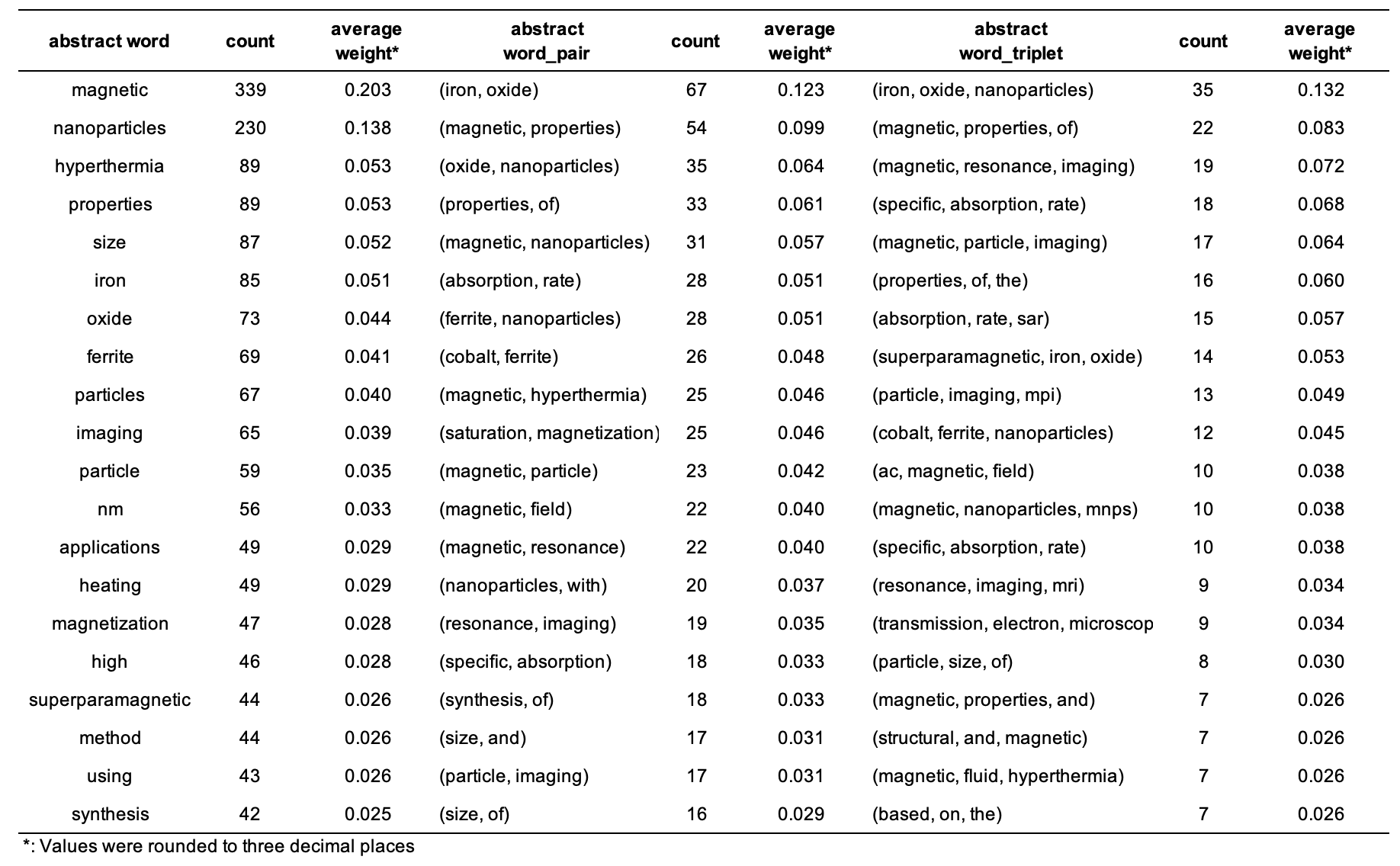


**Table S9.** Synonyms considered for feature searching.


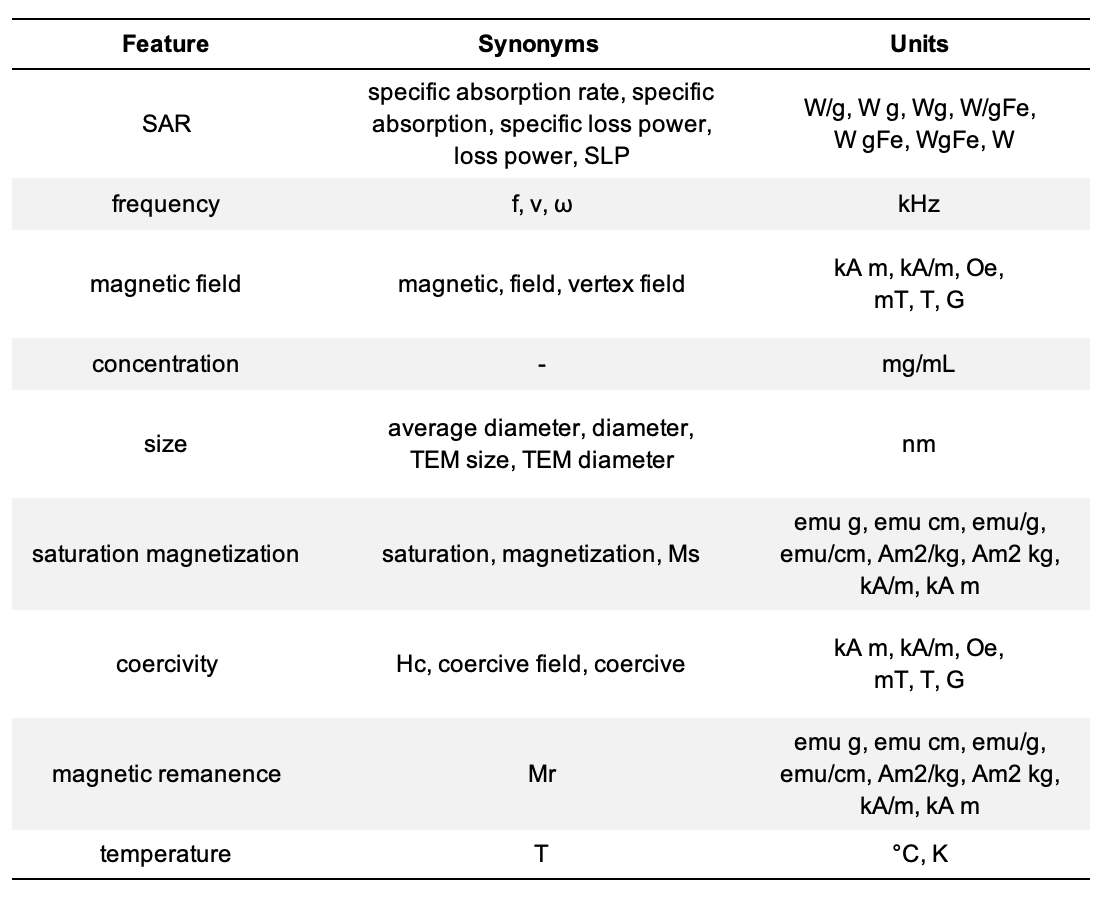


**Table S10.** Weighted RMSE metrics obtained through the calibration curve of the DNN model.


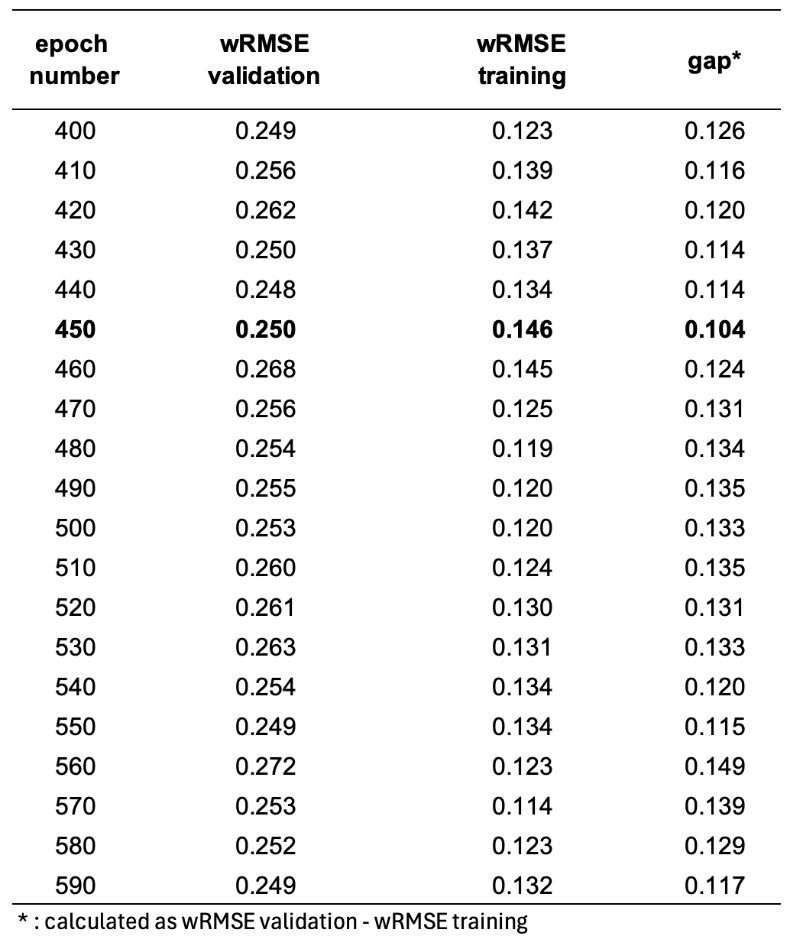

Supplement: Supplementary file 1 — Supporting File: smll72569‐sup‐0001‐SuppMat.docx. [file SMLL-22-e10453-s001.docx]
